# A neural speech decoding framework leveraging deep learning and speech synthesis

**DOI:** 10.1038/s42256-024-00824-8

**Published:** 2024-04-08

**Authors:** Xupeng Chen, Ran Wang, Amirhossein Khalilian-Gourtani, Leyao Yu, Patricia Dugan, Daniel Friedman, Werner Doyle, Orrin Devinsky, Yao Wang, Adeen Flinker

**Affiliations:** 1 Electrical and Computer Engineering Department, New York University, Brooklyn, NY USA; 2 Neurology Department, New York University, Manhattan, NY USA; 3 Biomedical Engineering Department, New York University, Brooklyn, NY USA; 4 Neurosurgery Department, New York University, Manhattan, NY USA

**Keywords:** Neural decoding, Cortex

## Abstract

Decoding human speech from neural signals is essential for brain–computer interface (BCI) technologies that aim to restore speech in populations with neurological deficits. However, it remains a highly challenging task, compounded by the scarce availability of neural signals with corresponding speech, data complexity and high dimensionality. Here we present a novel deep learning-based neural speech decoding framework that includes an ECoG decoder that translates electrocorticographic (ECoG) signals from the cortex into interpretable speech parameters and a novel differentiable speech synthesizer that maps speech parameters to spectrograms. We have developed a companion speech-to-speech auto-encoder consisting of a speech encoder and the same speech synthesizer to generate reference speech parameters to facilitate the ECoG decoder training. This framework generates natural-sounding speech and is highly reproducible across a cohort of 48 participants. Our experimental results show that our models can decode speech with high correlation, even when limited to only causal operations, which is necessary for adoption by real-time neural prostheses. Finally, we successfully decode speech in participants with either left or right hemisphere coverage, which could lead to speech prostheses in patients with deficits resulting from left hemisphere damage.

## Abstract (web summary)

Recent research has focused on restoring speech in populations with neurological deficits. Chen, Wang et al. develop a framework for decoding speech from neural signals, which could lead to innovative speech prostheses.

## Main

Speech loss due to neurological deficits is a severe disability that limits both work life and social life. Advances in machine learning and brain–computer interface (BCI) systems have pushed the envelope in the development of neural speech prostheses to enable people with speech loss to communicate^1–5^. An effective modality for acquiring data to develop such decoders involves electrocorticographic (ECoG) recordings obtained in patients undergoing epilepsy surgery^4–10^. Implanted electrodes in patients with epilepsy provide a rare opportunity to collect cortical data during speech with high spatial and temporal resolution, and such approaches have produced promising results in speech decoding^4,5,8–11^.

Two challenges are inherent to successfully carrying out speech decoding from neural signals. First, the data to train personalized neural-to-speech decoding models are limited in duration, and deep learning models require extensive training data. Second, speech production varies in rate, intonation, pitch and so on, even within a single speaker producing the same word, complicating the underlying model representation^12,13^. These challenges have led to diverse speech decoding approaches with a range of model architectures. Currently, public code to test and replicate findings across research groups is limited in availability.

Earlier approaches to decoding and synthesizing speech spectrograms from neural signals focused on linear models. These approaches achieved a Pearson correlation coefficient (PCC) of ~0.6 or lower, but with simple model architectures that are easy to interpret and do not require large training datasets^14–16^. Recent research has focused on deep neural networks leveraging convolutional^8,9^ and recurrent^5,10,17^ network architectures. These approaches vary across two major dimensions: the intermediate latent representation used to model speech and the speech quality produced after synthesis. For example, cortical activity has been decoded into an articulatory movement space, which is then transformed into speech, providing robust decoding performance but with a non-natural synthetic voice reconstruction^17^. Conversely, some approaches have produced naturalistic reconstruction leveraging wavenet vocoders^8^, generative adversarial networks (GAN)^11^ and unit selection^18^, but achieve limited accuracy. A recent study in one implanted patient^19^ provided both robust accuracies and a naturalistic speech waveform by leveraging quantized HuBERT features^20^ as an intermediate representation space and a pretrained speech synthesizer that converts the HuBERT features into speech. However, HuBERT features do not carry speaker-dependent acoustic information and can only be used to generate a generic speaker’s voice, so they require a separate model to translate the generic voice to a specific patient’s voice. Furthermore, this study and most previous approaches have employed non-causal architectures, which may limit real-time applications, which typically require causal operations.

**Fig. 1:**
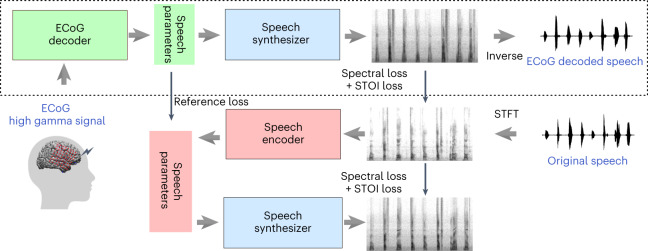
The proposed neural speech decoding framework. The upper part shows the ECoG-to-speech decoding pipeline. The ECoG decoder generates time-varying speech parameters from ECoG signals. The speech synthesizer generates spectrograms from the speech parameters. A separate spectrogram inversion algorithm converts the spectrograms to speech waveforms. The lower part shows the speech-to-speech auto-encoder, which generates the guidance for the speech parameters to be produced by the ECoG decoder during its training. The speech encoder maps an input spectrogram to the speech parameters, which are then fed to the same speech synthesizer to reproduce the spectrogram. The speech encoder and a few learnable subject-specific parameters in the speech synthesizer are pre-trained using speech signals only. Only the upper part is needed to decode the speech from ECoG signals once the pipeline is trained.

To address these issues, in this Article we present a novel ECoG-to-speech framework with a low-dimensional intermediate representation guided by subject-specific pre-training using speech signal only (Fig. 1). Our framework consists of an ECoG decoder that maps the ECoG signals to interpretable acoustic speech parameters (for example, pitch, voicing and formant frequencies), as well as a speech synthesizer that translates the speech parameters to a spectrogram. The speech synthesizer is differentiable, enabling us to minimize the spectrogram reconstruction error during training of the ECoG decoder. The low-dimensional latent space, together with guidance on the latent representation generated by a pre-trained speech encoder, overcomes data scarcity issues. Our publicly available framework produces naturalistic speech that highly resembles the speaker’s own voice, and the ECoG decoder can be realized with different deep learning model architectures and using different causality directions. We report this framework with multiple deep architectures (convolutional, recurrent and transformer) as the ECoG decoder, and apply it to 48 neurosurgical patients. Our framework performs with high accuracy across the models, with the best performance obtained by the convolutional (ResNet) architecture (PCC of 0.806 between the original and decoded spectrograms). Our framework can achieve high accuracy using only causal processing and relatively low spatial sampling on the cortex. We also show comparable speech decoding from grid implants on the left and right hemispheres, providing a proof of concept for neural prosthetics in patients suffering from expressive aphasia (with damage limited to the left hemisphere), although such an approach must be tested in patients with damage to the left hemisphere. Finally, we provide a publicly available neural decoding pipeline (https://github.com/flinkerlab/neural_speech_decoding) that offers flexibility in ECoG decoding architectures to push forward research across the speech science and prostheses communities.

## Results

### ECoG-to-speech decoding framework

Our ECoG-to-speech framework consists of an ECoG decoder and a speech synthesizer (shown in the upper part of Fig. 1). The neural signals are fed into an ECoG decoder, which generates speech parameters, followed by a speech synthesizer, which translates the parameters into spectrograms (which are then converted to a waveform by the Griffin–Lim algorithm^21^). The training of our framework comprises two steps. We first use semi-supervised learning on the speech signals alone. An auto-encoder, shown in the lower part of Fig. 1, is trained so that the speech encoder derives speech parameters from a given spectrogram, while the speech synthesizer (used here as the decoder) reproduces the spectrogram from the speech parameters. Our speech synthesizer is fully differentiable and generates speech through a weighted combination of voiced and unvoiced speech components generated from input time series of speech parameters, including pitch, formant frequencies, loudness and so on. The speech synthesizer has only a few subject-specific parameters, which are learned as part of the auto-encoder training (more details are provided in the Methods Speech synthesizer section). Currently, our speech encoder and speech synthesizer are subject-specific and can be trained using any speech signal of a participant, not just those with corresponding ECoG signals.

In the next step, we train the ECoG decoder in a supervised manner based on ground-truth spectrograms (using measures of spectrogram difference and short-time objective intelligibility, STOI^8,22^), as well as guidance for the speech parameters generated by the pre-trained speech encoder (that is, reference loss between speech parameters). By limiting the number of speech parameters (18 at each time step; Methods section Summary of speech parameters) and using the reference loss, the ECoG decoder can be trained with limited corresponding ECoG and speech data. Furthermore, because our speech synthesizer is differentiable, we can back-propagate the spectral loss (differences between the original and decoded spectrograms) to update the ECoG decoder. We provide multiple ECoG decoder architectures to choose from, including 3D ResNet^23^, 3D Swin Transformer^24^ and LSTM^25^. Importantly, unlike many methods in the literature, we employ ECoG decoders that can operate in a causal manner, which is necessary for real-time speech generation from neural signals. Note that, once the ECoG decoder and speech synthesizer are trained, they can be used for ECoG-to-speech decoding without using the speech encoder.

### Data collection

We employed our speech decoding framework across *N* = 48 participants who consented to complete a series of speech tasks (Methods section Experiments design). These participants, as part of their clinical care, were undergoing treatment for refractory epilepsy with implanted electrodes. During the hospital stay, we acquired synchronized neural and acoustic speech data. ECoG data were obtained from five participants with hybrid-density (HB) sampling (clinical-research grid) and 43 participants with low-density (LD) sampling (standard clinical grid), who took part in five speech tasks: auditory repetition (AR), auditory naming (AN), sentence completion (SC), word reading (WR) and picture naming (PN). These tasks were designed to elicit the same set of spoken words across tasks while varying the stimulus modality. We provided 50 repeated unique words (400 total trials per participant), all of which were analysed locked to the onset of speech production. We trained a model for each participant using 80% of available data for that participant and evaluated the model on the remaining 20% of data (with the exception of the more stringent word-level cross-validation).

### Speech decoding performance and causality

**Fig. 2:**
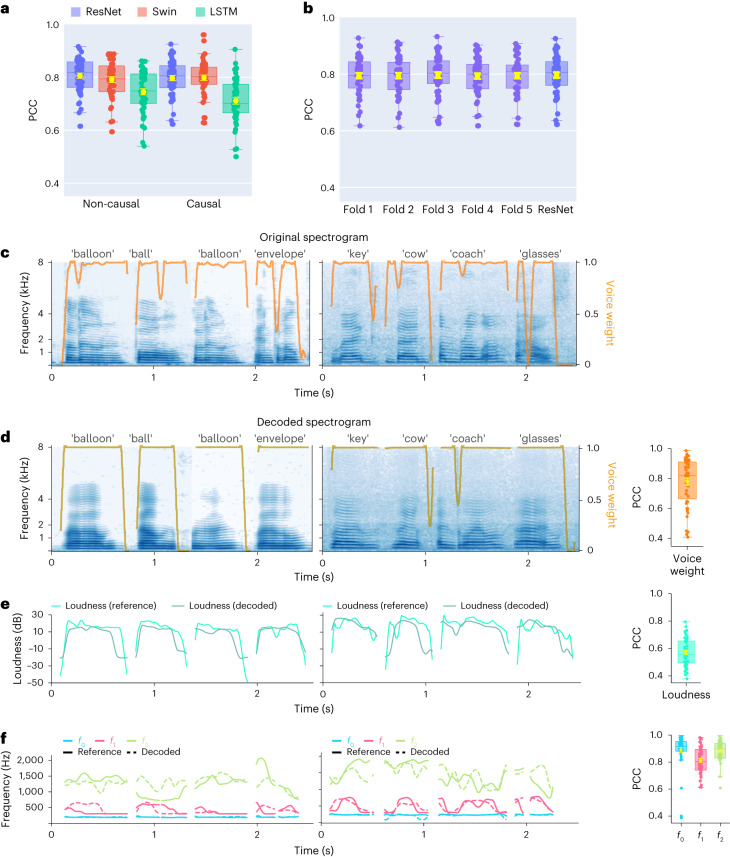
Decoding performance comparing the original and decoded spectrograms across non-causal and causal models. **a**, Performances of ResNet, Swin and LSTM models with non-causal and causal operations. The PCC between the original and decoded spectrograms is evaluated on the held-out testing set and shown for each participant. Each data point corresponds to a participant’s average PCC across testing trials. **b**, A stringent cross-validation showing the performance of the causal ResNet model on unseen words during training from five folds; we ensured that the training and validation sets in each fold did not overlap in unique words. The performance across all five validation folds was comparable to our trial-based validation, denoted for comparison as ResNet (identical to the ResNet causal model in **a**). **c**–**f**, Examples of decoded spectrograms and speech parameters from the causal ResNet model for eight words (from two participants) and the PCC values for the decoded and reference speech parameters across all participants. Spectrograms of the original (**c**) and decoded (**d**) speech are shown, with orange curves overlaid representing the reference voice weight learned by the speech encoder (**c**) and the decoded voice weight from the ECoG decoder (**d**). The PCC between the decoded and reference voice weights is shown on the right across all participants. **e**, Decoded and reference loudness parameters for the eight words, and the PCC values of the decoded loudness parameters across participants (boxplot on the right). **f**, Decoded (dashed) and reference (solid) parameters for pitch (*f*_0_) and the first two formants (*f*_1_ and *f*_2_) are shown for the eight words, as well as the PCC values across participants (box plots to the right). All box plots depict the median (horizontal line inside the box), 25th and 75th percentiles (box) and 25th or 75th percentiles ± 1.5 × interquartile range (whiskers) across all participants (*N* = 48). Yellow error bars denote the mean ± s.e.m. across participants. Source data

We first aimed to directly compare the decoding performance across different architectures, including those that have been employed in the neural speech decoding literature (recurrent and convolutional) and transformer-based models. Although any decoder architecture could be used for the ECoG decoder in our framework, employing the same speech encoder guidance and speech synthesizer, we focused on three representative models for convolution (ResNet), recurrent (LSTM) and transformer (Swin) architectures. Note that any of these models can be configured to use temporally non-causal or causal operations. Our results show that ResNet outperformed the other models, providing the highest PCC across *N* = 48 participants (mean PCC = 0.806 and 0.797 for non-causal and causal, respectively), closely followed by Swin (mean PCC = 0.792 and 0.798 for non-causal and causal, respectively) (Fig. 2a). We found the same when evaluating the three models using STOI+ (ref. ^26^), as shown in Supplementary Fig. 1a. The causality of machine learning models for speech production has important implications for BCI applications. A causal model only uses past and current neural signals to generate speech, whereas non-causal models use past, present and future neural signals. Previous reports have typically employed non-causal models^5,8,10,17^, which can use neural signals related to the auditory and speech feedback that is unavailable in real-time applications. Optimally, only the causal direction should be employed. We thus compared the performance of the same models with non-causal and causal temporal operations. Figure 2a compares the decoding results of causal and non-causal versions of our models. The causal ResNet model (PCC = 0.797) achieved a performance comparable to that of the non-causal model (PCC = 0.806), with no significant differences between the two (Wilcoxon two-sided signed-rank test *P* = 0.093). The same was true for the causal Swin model (PCC = 0.798) and its non-causal (PCC = 0.792) counterpart (Wilcoxon two-sided signed-rank test *P* = 0.196). In contrast, the performance of the causal LSTM model (PCC = 0.712) was significantly inferior to that of its non-causal (PCC = 0.745) version (Wilcoxon two-sided signed-rank test *P* = 0.009). Furthermore, the LSTM model showed consistently lower performance than ResNet and Swin. However, we did not find significant differences between the causal ResNet and causal Swin performances (Wilcoxon two-sided signed-rank test *P* = 0.587). Because the ResNet and Swin models had the highest performance and were on par with each other and their causal counterparts, we chose to focus further analyses on these causal models, which we believe are best suited for prosthetic applications.

To ensure our framework can generalize well to unseen words, we added a more stringent word-level cross-validation in which random (ten unique) words were entirely held out during training (including both pre-training of the speech encoder and speech synthesizer and training of the ECoG decoder). This ensured that different trials from the same word could not appear in both the training and testing sets. The results shown in Fig. 2b demonstrate that performance on the held-out words is comparable to our standard trial-based held-out approach (Fig. 2a, ‘ResNet’). It is encouraging that the model can decode unseen validation words well, regardless of which words were held out during training.

Next, we show the performance of the ResNet causal decoder on the level of single words across two representative participants (LD grids). The decoded spectrograms accurately preserve the spectro-temporal structure of the original speech (Fig. 2c,d). We also compare the decoded speech parameters with the reference parameters. For each parameter, we calculated the PCC between the decoded time series and the reference sequence, showing average PCC values of 0.781 (voice weight, Fig. 2d), 0.571 (loudness, Fig. 2e), 0.889 (pitch *f*_0_, Fig. 2f), 0.812 (first formant *f*_1_, Fig. 2f) and 0.883 (second formant *f*_2_, Fig. 2f). Accurate reconstruction of the speech parameters, especially the pitch, voice weight and first two formants, is essential for accurate speech decoding and naturalistic reconstruction that mimics a participant’s voice. We also provide a non-causal version of Fig. 2 in Supplementary Fig. 2. The fact that both non-causal and causal models can yield reasonable decoding results is encouraging.

### Left-hemisphere versus right-hemisphere decoding

**Fig. 3:**
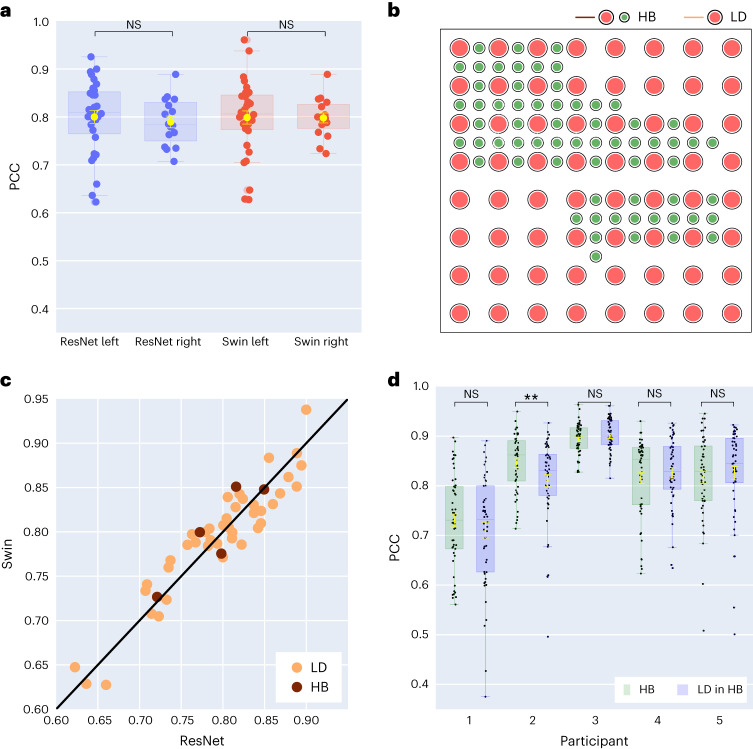
Comparison of decoding performance under different settings of the 3D ResNet and 3D Swin models. **a**, Comparison between left- and right-hemisphere participants using causal models. No statistically significant differences (ResNet independent *t*-test, *P* = 0.623; Swin Wilcoxon independent *t*-test, *P* = 0.968) in PCC values exist between left- (*N* = 32) and right- (*N* = 16) hemisphere participants. **b**, An example hybrid-density ECoG array with a total of 128 electrodes. The 64 electrodes marked in red correspond to a LD placement. The remaining 64 green electrodes, combined with red electrodes, reflect HB placement. **c**, Comparison between causal ResNet and causal Swin models for the same participant across participants with HB (*N* = 5) or LD (*N* = 43) ECoG grids. The two models show similar decoding performances from the HB and LD grids. **d**, Decoding PCC values across 50 test trials by the ResNet model for HB (*N* = 5) participants when all electrodes are used versus when only LD-in-HB electrodes (*N* = 5) are considered. There are no statistically significant differences for four out of five participants (Wilcoxon two-sided signed-rank test, *P* = 0.114, 0.003, 0.0773, 0.472 and 0.605, respectively). All box plots depict the median (horizontal line inside box), 25th and 75th percentiles (box) and 25th or 75th percentiles ± 1.5 × interquartile range (whiskers). Yellow error bars denote mean ± s.e.m. Distributions were compared with each other as indicated, using the Wilcoxon two-sided signed-rank test and independent *t*-test. **P < 0.01; NS, not significant. Source data

Most speech decoding studies have focused on the language- and speech-dominant left hemisphere^27^. However, little is known about decoding speech representations from the right hemisphere. To this end, we compared left- versus right-hemisphere decoding performance across our participants to establish the feasibility of a right-hemisphere speech prosthetic. For both our ResNet and Swin decoders, we found robust speech decoding from the right hemisphere (ResNet PCC = 0.790, Swin PCC = 0.798) that was not significantly different from that of the left (Fig. 3a, ResNet independent *t*-test, *P* = 0.623; Swin independent *t*-test, *P* = 0.968). A similar conclusion held when evaluating STOI+ (Supplementary Fig. 1b, ResNet independent *t*-test, *P* = 0.166; Swin independent *t*-test, *P* = 0.114). Although these results suggest that it may be feasible to use neural signals in the right hemisphere to decode speech for patients who suffer damage to the left hemisphere and are unable to speak^28^, it remains unknown whether intact left-hemisphere cortex is necessary to allow for speech decoding from the right hemisphere until tested in such patients.

### Effect of electrode density

Next, we assessed the impact of electrode sampling density on speech decoding, as many previous reports use higher-density grids (0.4 mm) with more closely spaced contacts than typical clinical grids (1 cm). Five participants consented to hybrid grids (Fig. 3b, HB), which typically had LD electrode sampling but with additional electrodes interleaved. The HB grids provided a decoding performance similar to clinical LD grids in terms of PCC values (Fig. 3c), with a slight advantage in STOI+, as shown in Supplementary Fig. 3b. To ascertain whether the additional spatial sampling indeed provides improved speech decoding, we compared models that decode speech based on all the hybrid electrodes versus only the LD electrodes in participants with HB grids (comparable to our other LD participants). Our findings (Fig. 3d) suggest that the decoding results were not significantly different from each other (with the exception of participant 2) in terms of PCC and STOI+ (Supplementary Fig. 3c). Together, these results suggest that our models can learn speech representations well from both high and low spatial sampling of the cortex, with the exciting finding of robust speech decoding from the right hemisphere.

### Contribution analysis

**Fig. 4:**
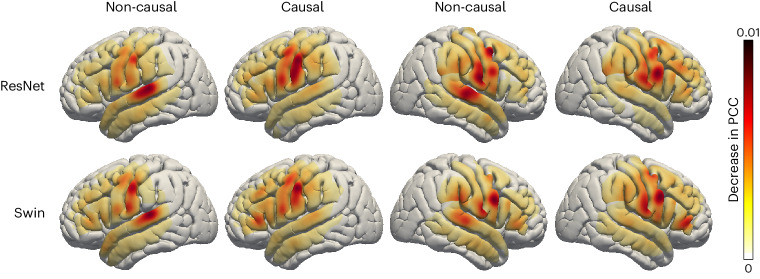
Contribution analysis. Visualization of the contribution of each cortical location to the decoding result achieved by both causal and non-causal decoding models through an occlusion analysis. The contribution of each electrode region in each participant is projected onto the standardized Montreal Neurological Institute (MNI) brain anatomical map and then averaged over all participants. Each subplot shows the causal or non-causal contribution of different cortical locations (red indicates a higher contribution; yellow indicates a lower contribution). For visualization purposes, we normalized the contribution of each electrode location by the local grid density, because there were multiple participants with non-uniform density.

Finally, we investigated which cortical regions contribute to decoding to provide insight for the targeted implantation of future prosthetics, especially on the right hemisphere, which has not yet been investigated. We used an occlusion approach to quantify the contributions of different cortical sites to speech decoding. If a region is involved in decoding, occluding the neural signal in the corresponding electrode (that is, setting the signal to zero) will reduce the accuracy (PCC) of the speech reconstructed on testing data (Methods section Contribution analysis). We thus measured each region’s contribution by decoding the reduction in the PCC when the corresponding electrode was occluded. We analysed all electrodes and participants with causal and non-causal versions of the ResNet and Swin decoders. The results in Fig. 4 show similar contributions for the ResNet and Swin models (Supplementary Figs. 8 and 9 describe the noise-level contribution). The non-causal models show enhanced auditory cortex contributions compared with the causal models, implicating auditory feedback in decoding, and underlying the importance of employing only causal models during speech decoding because neural feedback signals are not available for real-time decoding applications. Furthermore, across the causal models, both the right and left hemispheres show similar contributions across the sensorimotor cortex, especially on the ventral portion, suggesting the potential feasibility of right-hemisphere neural prosthetics.

## Discussion

**Fig. 5:**
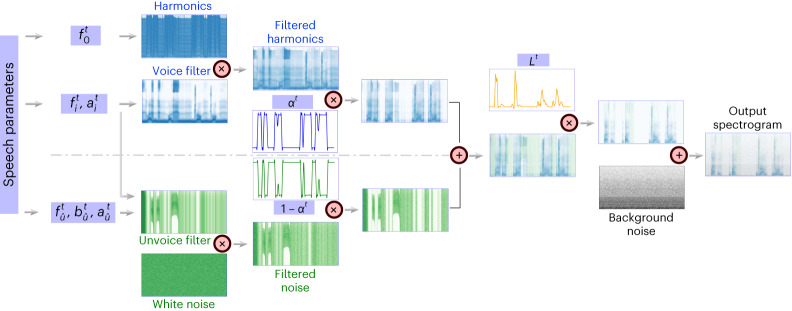
Differentiable speech synthesizer architecture. Our speech synthesizer generates the spectrogram at time *t* by combining a voiced component and an unvoiced component based on a set of speech parameters at *t*. The upper part represents the voice pathway, which generates the voiced component by passing a harmonic excitation with fundamental frequency ${f}_{0}^{\;t}$ through a voice filter (which is the sum of six formant filters, each specified by formant frequency ${f}_{i}^{\;t}$ and amplitude ${a}_{i}^{t}$). The lower part describes the noise pathway, which synthesizes the unvoiced sound by passing white noise through an unvoice filter (consisting of a broadband filter defined by centre frequency ${f}_{\hat{u}}^{\;t}$, bandwidth ${b}_{\hat{u}}^{t}$ and amplitude ${a}_{\hat{u}}^{t}$, and the same six formant filters used for the voice filter). The two components are next mixed with voice weight *α*^*t*^ and unvoice weight 1 − *α*^*t*^, respectively, and then amplified by loudness *L*^*t*^. A background noise (defined by a stationary spectrogram *B*(*f*)) is finally added to generate the output spectrogram. There are a total of 18 speech parameters at any time *t*, indicated in purple boxes.

**Fig. 6:**
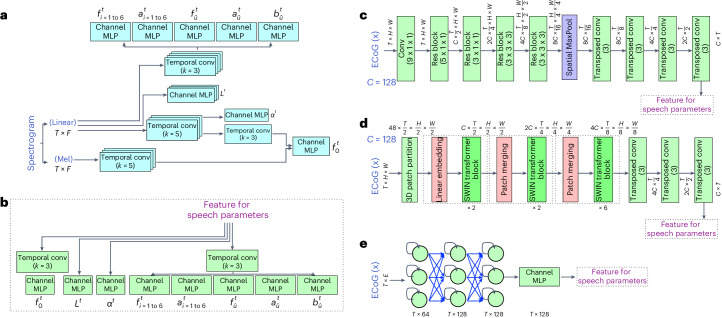
Speech encoder and ECoG decoder. **a**, The speech encoder architecture. We input a spectrogram into a network of temporal convolution layers and channel MLPs that produce speech parameters. **b**,**c**, The ECoG decoder (**c**) using the 3D ResNet architecture. We first use several temporal and spatial convolutional layers with residual connections and spatiotemporal pooling to generate downsampled latent features, and then use corresponding transposed temporal convolutional layers to upsample the features to the original temporal dimension. We then apply temporal convolution layers and channel MLPs to map the features to speech parameters, as shown in **b**. The non-causal version uses non-causal temporal convolution in each layer, whereas the causal version uses causal convolution. **d**, The ECoG decoder using the 3D Swin architecture. We use three or four stages of 3D Swin blocks with spatial-temporal attention (three blocks for LD and four blocks for HB) to extract the features from the ECoG signal. We then use the transposed versions of temporal convolution layers as in **c** to upsample the features. The resulting features are mapped to the speech parameters using the same structure as shown in **b**. Non-causal versions apply temporal attention to past, present and future tokens, whereas the causal version applies temporal attention only to past and present tokens. **e**, The ECoG decoder using LSTM layers. We use three LSTM layers and one layer of channel MLP to generate features. We then reuse the prediction layers in **b** to generate the corresponding speech parameters. The non-causal version employs bidirectional LSTM in each layer, whereas the causal version uses unidirectional LSTM.

Our novel pipeline can decode speech from neural signals by leveraging interchangeable architectures for the ECoG decoder and a novel differentiable speech synthesizer (Fig. 5). Our training process relies on estimating guidance speech parameters from the participants’ speech using a pre-trained speech encoder (Fig. 6a). This strategy enabled us to train ECoG decoders with limited corresponding speech and neural data, which can produce natural-sounding speech when paired with our speech synthesizer. Our approach was highly reproducible across participants (*N* = 48), providing evidence for successful causal decoding with convolutional (ResNet; Fig. 6c) and transformer (Swin; Fig. 6d) architectures, both of which outperformed the recurrent architecture (LSTM; Fig. 6e). Our framework can successfully decode from both high and low spatial sampling with high levels of decoding performance. Finally, we provide potential evidence for robust speech decoding from the right hemisphere as well as the spatial contribution of cortical structures to decoding across the hemispheres.

Our decoding pipeline showed robust speech decoding across participants, leading to PCC values within the range 0.62–0.92 (Fig. 2a; causal ResNet mean 0.797, median 0.805) between the decoded and ground-truth speech across several architectures. We attribute our stable training and accurate decoding to the carefully designed components of our pipeline (for example, the speech synthesizer and speech parameter guidance) and the multiple improvements (Methods sections Speech synthesizer, ECoG decoder and Model training) over our previous approach on the subset of participants with hybrid-density grids^29^. Previous reports have investigated speech- or text-decoding using linear models^14,15,30^, transitional probability^4,31^, recurrent neural networks^5,10,17,19^, convolutional neural networks^8,29^ and other hybrid or selection approaches^9,16,18,32,33^. Overall, our results are similar to (or better than) many previous reports (54% of our participants showed higher than 0.8 for the decoding PCC; Fig. 3c). However, a direct comparison is complicated by multiple factors. Previous reports vary in terms of the reported performance metrics, as well as the stimuli decoded (for example, continuous speech versus single words) and the cortical sampling (that is, high versus low density, depth electrodes compared with surface grids). Our publicly available pipeline, which can be used across multiple neural network architectures and tested on various performance metrics, can facilitate the research community to conduct more direct comparisons while still adhering to a high accuracy of speech decoding.

The temporal causality of decoding operations, critical for real-time BCI applications, has not been considered by most previous studies. Many of these non-causal models relied on auditory (and somatosensory) feedback signals. Our analyses show that non-causal models rely on a robust contribution from the superior temporal gyrus (STG), which is mostly eliminated using a causal model (Fig. 4). We believe that non-causal models would show limited generalizability to real-time BCI applications due to their over-reliance on feedback signals, which may be absent (if no delay is allowed) or incorrect (if a short latency is allowed during real-time decoding). Some approaches used imagined speech, which avoids feedback during training^16^, or showed generalizability to mimed production lacking auditory feedback^17,19^. However, most reports still employ non-causal models, which cannot rule out feedback during training and inference. Indeed, our contribution maps show robust auditory cortex recruitment for the non-causal ResNet and Swin models (Fig. 4, in contrast to their causal counterparts, which decode based on more frontal regions. Furthermore, the recurrent neural networks that are widely used in the literature^5,19^ are typically bidirectional, producing non-causal behaviours and longer latencies for prediction during real-time applications. Unidirectional causal results are typically not reported. The recurrent network we tested performed the worst when trained with one direction (Fig. 2a, causal LSTM). Although our current focus was not real-time decoding, we were able to synthesize speech from neural signals with a delay of under 50 ms (Supplementary Table 1), which provides minimal auditory delay interference and allows for normal speech production^34,35^. Our data suggest that causal convolutional and transformer models can perform on par with their non-causal counterparts and recruit more relevant cortical structures for real-time decoding.

In our study we have leveraged an intermediate speech parameter space together with a novel differentiable speech synthesizer to decode subject-specific naturalistic speech (Fig. 1. Previous reports used varying approaches to model speech, including an intermediate kinematic space^17^, an acoustically relevant intermediate space using HuBERT features^19^ derived from a self-supervised speech masked prediction task^20^, an intermediate random vector (that is, GAN)^11^ or direct spectrogram representations^8,17,36,37^. Our choice of speech parameters as the intermediate representation allowed us to decode subject-specific acoustics. Our intermediate acoustic representation led to significantly more accurate speech decoding than directly mapping ECoG to the speech spectrogram^38^, and than mapping ECoG to a random vector, which is then fed to a GAN-based speech synthesizer^11^ (Supplementary Fig. 10). Unlike the kinematic representation, our acoustic intermediate representation using speech parameters and the associated speech synthesizer enables our decoding pipeline to produce natural-sounding speech that preserves subject-specific characteristics, which would be lost with the kinematic representation.

Our speech synthesizer is motivated by classical vocoder models for speech production (generating speech by passing an excitation source, harmonic or noise, through a filter^39,40^ and is fully differentiable, facilitating the training of the ECoG decoder using spectral losses through backpropagation. Furthermore, the guidance speech parameters needed for training the ECoG decoder can be obtained using a speech encoder that can be pre-trained without requiring neural data. Thus, it could be trained using older speech recordings or a proxy speaker chosen by the patient in the case of patients without the ability to speak. Training the ECoG decoder using such guidance, however, would require us to revise our current training strategy to overcome the challenge of misalignment between neural signals and speech signals, which is a scope of our future work. Additionally, the low-dimensional acoustic space and pre-trained speech encoder (for generating the guidance) using speech signals only alleviate the limited data challenge in training the ECoG-to-speech decoder and provide a highly interpretable latent space. Finally, our decoding pipeline is generalizable to unseen words (Fig. 2b). This provides an advantage compared to the pattern-matching approaches^18^ that produce subject-specific utterances but with limited generalizability.

Many earlier studies employed high-density electrode coverage over the cortex, providing many distinct neural signals^5,10,17,30,37^. One question we directly addressed was whether higher-density coverage improves decoding. Surprisingly, we found a high decoding performance in terms of spectrogram PCC with both low-density and higher (hybrid) density grid coverages (Fig. 3c). Furthermore, comparing the decoding performance obtained using all electrodes in our hybrid-density participants versus using only the low-density electrodes in the same participants revealed that the decoding did not differ significantly (albeit for one participant; Fig. 3d). We attribute these results to the ability of our ECoG decoder to extract speech parameters from neural signals as long as there is sufficient perisylvian coverage, even in low-density participants.

A striking result was the robust decoding from right hemisphere cortical structures as well as the clear contribution of the right perisylvian cortex. Our results are consistent with the idea that syllable-level speech information is represented bilaterally^41^. However, our findings suggest that speech information is well-represented in the right hemisphere. Our decoding results could directly lead to speech prostheses for patients who suffer from expressive aphasia or apraxia of speech. Some previous studies have shown limited right-hemisphere decoding of vowels^42^ and sentences^43^. However, the results were mostly mixed with left-hemisphere signals. Although our decoding results provide evidence for a robust representation of speech in the right hemisphere, it is important to note that these regions are likely not critical for speech, as evidenced by the few studies that have probed both hemispheres using electrical stimulation mapping^44,45^. Furthermore, it is unclear whether the right hemisphere would contain sufficient information for speech decoding if the left hemisphere were damaged. It would be necessary to collect right-hemisphere neural data from left-hemisphere-damaged patients to verify we can still achieve acceptable speech decoding. However, we believe that right-hemisphere decoding is still an exciting avenue as a clinical target for patients who are unable to speak due to left-hemisphere cortical damage.

There are several limitations in our study. First, our decoding pipeline requires speech training data paired with ECoG recordings, which may not exist for paralysed patients. This could be mitigated by using neural recordings during imagined or mimed speech and the corresponding older speech recordings of the patient or speech by a proxy speaker chosen by the patient. As discussed earlier, we would need to revise our training strategy to overcome the temporal misalignment between the neural signal and the speech signal. Second, our ECoG decoder models (3D ResNet and 3D Swin) assume a grid-based electrode sampling, which may not be the case. Future work should develop model architectures that are capable of handling non-grid data, such as strips and depth electrodes (stereo intracranial electroencephalogram (sEEG)). Importantly, such decoders could replace our current grid-based ECoG decoders while still being trained using our overall pipeline. Finally, our focus in this study was on word-level decoding limited to a vocabulary of 50 words, which may not be directly comparable to sentence-level decoding. Specifically, two recent studies have provided robust speech decoding in a few chronic patients implanted with intracranial ECoG^19^ or a Utah array^46^ that leveraged a large amount of data available in one patient in each study. It is noteworthy that these studies use a range of approaches in constraining their neural predictions. Metzger and colleagues employed a pre-trained large transformer model leveraging directional attention to provide the guidance HuBERT features for their ECoG decoder. In contrast, Willet and colleagues decoded at the level of phonemes and used transition probability models at both phoneme and word levels to constrain decoding. Our study is much more limited in terms of data. However, we were able to achieve good decoding results across a large cohort of patients through the use of a compact acoustic representation (rather than learnt contextual information). We expect that our approach can help improve generalizability for chronically implanted patients.

To summarize, our neural decoding approach, capable of decoding natural-sounding speech from 48 participants, provides the following major contributions. First, our proposed intermediate representation uses explicit speech parameters and a novel differentiable speech synthesizer, which enables interpretable and acoustically accurate speech decoding. Second, we directly consider the causality of the ECoG decoder, providing strong support for causal decoding, which is essential for real-time BCI applications. Third, our promising decoding results using low sampling density and right-hemisphere electrodes shed light on future neural prosthetic devices using low-density grids and in patients with damage to the left hemisphere. Last, but not least, we have made our decoding framework open to the community with documentation (https://github.com/flinkerlab/neural_speech_decoding), and we trust that this open platform will help propel the field forward, supporting reproducible science.

## Methods

### Experiments design

We collected neural data from 48 native English-speaking participants (26 female, 22 male) with refractory epilepsy who had ECoG subdural electrode grids implanted at NYU Langone Hospital. Five participants underwent HB sampling, and 43 LD sampling. The ECoG array was implanted on the left hemisphere for 32 participants and on the right for 16. The Institutional Review Board of NYU Grossman School of Medicine approved all experimental procedures. After consulting with the clinical-care provider, a research team member obtained written and oral consent from each participant. Each participant performed five tasks^47^ to produce target words in response to auditory or visual stimuli. The tasks were auditory repetition (AR, repeating auditory words), auditory naming (AN, naming a word based on an auditory definition), sentence completion (SC, completing the last word of an auditory sentence), visual reading (VR, reading aloud written words) and picture naming (PN, naming a word based on a colour drawing).

For each task, we used the exact 50 target words with different stimulus modalities (auditory, visual and so on). Each word appeared once in the AN and SC tasks and twice in the others. The five tasks involved 400 trials, with corresponding word production and ECoG recording for each participant. The average duration of the produced speech in each trial was 500 ms.

### Data collection and preprocessing

The study recorded ECoG signals from the perisylvian cortex (including STG, inferior frontal gyrus (IFG), pre-central and postcentral gyri) of 48 participants while they performed five speech tasks. A microphone recorded the subjects’ speech and was synchronized to the clinical Neuroworks Quantum Amplifier (Natus Biomedical), which captured ECoG signals. The ECoG array consisted of 64 standard 8 × 8 macro contacts (10-mm spacing) for 43 participants with low-density sampling. For five participants with hybrid-density sampling, the ECoG array also included 64 additional interspersed smaller electrodes (1 mm) between the macro contacts (providing 10-mm centre-to-centre spacing between macro contacts and 5-mm centre-to-centre spacing between micro/macro contacts; PMT Corporation) (Fig. 3b). This Food and Drug Administration (FDA)-approved array was manufactured for this study. A research team member informed participants that the additional contacts were for research purposes during consent. Clinical care solely determined the placement location across participants (32 left hemispheres; 16 right hemispheres). The decoding models were trained separately for each participant using all trials except ten randomly selected ones from each task, leading to 350 trials for training and 50 for testing. The reported results are for testing data only.

We sampled ECoG signals from each electrode at 2,048 Hz and downsampled them to 512 Hz before processing. Electrodes with artefacts (for example, line noise, poor contact with the cortex, high-amplitude shifts) were rejected. The electrodes with interictal and epileptiform activity were also excluded from the analysis. The mean of a common average reference (across all remaining valid electrodes and time) was subtracted from each individual electrode. After the subtraction, a Hilbert transform extracted the envelope of the high gamma (70–150 Hz) component from the raw signal, which was then downsampled to 125 Hz. A reference signal was obtained by extracting a silent period of 250 ms before each trial’s stimulus period within the training set and averaging the signals over these silent periods. Each electrode’s signal was normalized to the reference mean and variance (that is, *z*-score). The data-preprocessing pipeline was coded in MATLAB and Python. For participants with noisy speech recordings, we applied spectral gating to remove stationary noise from the speech using an open-source tool^48^. We ruled out the possibility that our neural data suffer from a recently reported acoustic contamination (Supplementary Fig. 5) by following published approaches^49^.

To pre-train the auto-encoder, including the speech encoder and speech synthesizer, unlike our previous work in ref. ^29^, which completely relied on unsupervised training, we provided supervision for some speech parameters to improve their estimation accuracy further. Specifically, we used the Praat method^50^ to estimate the pitch and four formant frequencies (${f}_{ {{{\rm{i}}}} = {1}\,{{{\rm{to}}}}\,4}^{t}$, in hertz) from the speech waveform. The estimated pitch and formant frequency were resampled to 125 Hz, the same as the ECoG signal and spectrogram sampling frequency. The mean square error between these speech parameters generated by the speech encoder and those estimated by the Praat method was used as a supervised reference loss, in addition to the unsupervised spectrogram reconstruction and STOI losses, making the training of the auto-encoder semi-supervised.

### Speech synthesizer

Our speech synthesizer was inspired by the traditional speech vocoder, which generates speech by switching between voiced and unvoiced content, each generated by filtering a specific excitation signal. Instead of switching between the two components, we use a soft mix of the two components, making the speech synthesizer differentiable. This enables us to train the ECoG decoder and the speech encoder end-to-end by minimizing the spectrogram reconstruction loss with backpropagation. Our speech synthesizer can generate a spectrogram from a compact set of speech parameters, enabling training of the ECoG decoder with limited data. As shown in Fig. 5, the synthesizer takes dynamic speech parameters as input and contains two pathways. The voice pathway applies a set of formant filters (each specified by the centre frequency ${f}_{i}^{\;t}$, bandwidth ${b}_{i}^{t}$ that is dependent on ${f}_{i}^{\;t}$, and amplitude ${a}_{i}^{t}$) to the harmonic excitation (with pitch frequency *f*_0_) and generates the voiced component, *V*^*t*^(*f*), for each time step *t* and frequency *f*. The noise pathway filters the input white noise with an unvoice filter (consisting of a broadband filter defined by centre frequency ${f}_{\hat{u}}^{\;t}$, bandwidth ${b}_{\hat{u}}^{t}$ and amplitude ${a}_{\hat{u}}^{t}$ and the same six formant filters used for the voice filter) and produces the unvoiced content, *U*^*t*^(*f*). The synthesizer combines the two components with a voice weight *α*^*t*^ ∈ [0, 1] to obtain the combined spectrogram ${\widetilde{S}}^{t}{(\;f\;)}$ as

$${\widetilde{S}}^{t}{(\;f\;)}={\alpha }^{t}{V}^{\;t}{(\;f\;)}+{(1-{\alpha }^{t}){U}^{\;t}(\;f\;)}$$

Factor *α*^*t*^ acts as a soft switch for the gradient to flow back through the synthesizer. The final speech spectrogram is given by

$${\widehat{S}}^{t}{(\;f\;)}={L}^{t}{\widetilde{S}}^{t}{(\;f\;)+B(\;f\;)}$$

where *L*^*t*^ is the loudness modulation and *B*(*f*) the background noise. We describe the various components in more detail in the following.

#### Formant filters in the voice pathway

We use multiple formant filters in the voice pathway to model formants that represent vowels and nasal information. The formant filters capture the resonance in the vocal tract, which can help recover a speaker’s timbre characteristics and generate natural-sounding speech. We assume the filter for each formant is time-varying and can be derived from a prototype filter *G*_*i*_(*f*), which achieves maximum at a centre frequency ${f}_{i}^{{{\;{\rm{proto}}}}}$ and has a half-power bandwidth ${b}_{i}^{{{{\rm{proto}}}}}$. The prototype filters have learnable parameters and will be discussed later. The actual formant filter at any time is written as a shifted and scaled version of *G*_*i*_(*f*). Specifically, at time *t*, given an amplitude ${\left({a}_{i}^{t}\right)}$, centre frequency ${\left(\;{f}_{i}^{\;t}\right)}$ and bandwidth ${\left({b}_{i}^{t}\right)}$, the frequency-domain representation of the *i*th formant filter is

1 $${F}_{i}^{\;t}{(\;f\;)}={a}_{i}^{t}\times {G}_{i}\left(\frac{{b}_{i}^{{{{\rm{proto}}}}}}{{b}_{i}^{t}}\times \left(\,{f}-{f}_{i}^{\;t}\right)+{f}_{i}^{{{\;{\rm{proto}}}}}\right),\,{f}\in [{0},\,{f}_{\max }]$$

where *f*_max_ is half of the speech sampling frequency, which in our case is 8,000 Hz.

Rather than letting the bandwidth parameters ${b}_{i}^{t}$ be independent variables, based on the empirically observed relationships between ${b}_{i}^{t}$ and the centre frequencies ${f}_{i}^{\;t}$, we set

2 $${b}_{i}^{t}=\left\{\begin{array}{ll}{a}\left(\;{f}_{i}^{\;t}-{f}_{\theta }\right)+{b}_{0},\quad &\,{{\rm{if}}}\,{f}_{i}^{\;t} > {f}_{\theta }\\ {b}_{0},\quad &\,{{\rm{otherwise}}}\,\end{array}\right.$$

The threshold frequency *f*_*θ*_, slope *a* and baseline bandwidth *b*_0_ are three parameters that are learned during the auto-encoder training, shared among all six formant filters. This parameterization helps to reduce the number of speech parameters to be estimated at every time sample, making the representation space more compact.

Finally the filter for the voice pathway with *N* formant filters is given by ${F}_{{{{\rm{v}}}}}^{\;t}{(\;f\;)}={\mathop{\sum }\nolimits_{i = 1}^{N}{F}_{i}^{\;t}(\;f\;)}$. Previous studies have shown that two formants (*N* = 2) are enough for intelligible reconstruction^51^, but we use *N* = 6 for more accurate synthesis in our experiments.

#### Unvoice filters

We construct the unvoice filter by adding a single broadband filter ${F}_{\hat{u}}^{\;t}{(\;f\;)}$ to the formant filters for each time step *t*. The broadband filter ${F}_{\hat{u}}^{\;t}{(\;f\;)}$ has the same form as equation (1) but has its own learned prototype filter ${G}_{\hat{u}}{(f)}$. The speech parameters corresponding to the broadband filter include ${\left({\alpha }_{\hat{u}}^{t},\,{f}_{\hat{u}}^{\;t},\,{b}_{\hat{u}}^{t}\right)}$. We do not impose a relationship between the centre frequency ${f}_{\hat{u}}^{\;t}$ and the bandwidth ${b}_{\hat{u}}^{t}$. This allows more flexibility in shaping the broadband unvoice filter. However, we constrain ${b}_{\hat{u}}^{t}$ to be larger than 2,000 Hz to capture the wide spectral range of obstruent phonemes. Instead of using only the broadband filter, we also retain the *N* formant filters in the voice pathway ${F}_{i}^{\;t}$ for the noise pathway. This is based on the observation that humans perceive consonants such as /p/ and /d/ not only by their initial bursts but also by their subsequent formant transitions until the next vowel^52^. We use identical formant filter parameters to encode these transitions. The overall unvoice filter is ${F}_{{{{\rm{u}}}}}^{\;t}{(\;f\;)}={F}_{\hat{u}}^{\;t}(\;f\;)+\mathop{\sum }\nolimits_{i = 1}^{N}{F}_{i}^{\;t}{(\;f\;)}$.

#### Voice excitation

We use the voice filter in the voice pathway to modulate the harmonic excitation. Following ref. ^53^, we define the harmonic excitation as ${h}^{t}={\mathop{\sum }\nolimits_{k = 1}^{K}{h}_{k}^{t}}$, where *K* = 80 is the number of harmonics.

The value of the *k*th resonance at time step *t* is ${h}_{k}^{t}={\sin (2\uppi k{\phi }^{t})}$ with ${\phi }^{t}={\mathop{\sum }\nolimits_{\tau = 0}^{t}{f}_{0}^{\;\tau }}$, where ${f}_{0}^{\;\tau }$ is the fundamental frequency at time *τ*. The spectrogram of *h*^*t*^ forms the harmonic excitation in the frequency domain *H*^*t*^(*f*), and the voice excitation is ${V}^{\;t}{(\;f\;)}={F}_{{{{\rm{v}}}}}^{t}{(\;f\;)}{H}^{\;t}{(\;f\;)}$.

#### Noise excitation

The noise pathway models consonant sounds (plosives and fricatives). It is generated by passing a stationary Gaussian white noise excitation through the unvoice filter. We first generate the noise signal *n*(*t*) in the time domain by sampling from the Gaussian process ${{{\mathcal{N}}}}{(0,\,1)}$ and then obtain its spectrogram *N*^*t*^(*f*). The spectrogram of the unvoiced component is ${U}^{\;t}{(\;f\;)}={F}_{u}^{\;t}{(\;f\;)}{N}^{\;t}{(\;f\;)}$.

#### Summary of speech parameters

The synthesizer generates the voiced component at time *t* by driving a harmonic excitation with pitch frequency ${f}_{0}^{\;t}$ through *N* formant filters in the voice pathway, each described by two parameters (${f}_{ i}^{\;t},\,{a}_{ i}^{t}$). The unvoiced component is generated by filtering a white noise through the unvoice filter consisting of an additional broadband filter with three parameters (${f}_{\hat{u}}^{\;t},\,{b}_{\hat{u}}^{t},\,{a}_{\hat{u}}^{t}$). The two components are mixed based on the voice weight *α*^*t*^ and further amplified by the loudness value *L*^*t*^. In total, the synthesizer input includes 18 speech parameters at each time step.

Unlike the differentiable digital signal processing (DDSP) in ref. ^53^, we do not directly assign amplitudes to the *K* harmonics. Instead, the amplitude in our model depends on the formant filters, which has two benefits:

- The representation space is more compact. DDSP requires 80 amplitude parameters ${a}_{k}^{t}$ for each of the 80 harmonic components ${f}_{k}^{\;t}$ (*k* = 1, 2, …, 80) at each time step. In contrast, our synthesizer only needs a total of 18 parameters.
- The representation is more disentangled. For human speech, the vocal tract shape (affecting the formant filters) is largely independent of the vocal cord tension (which determines the pitch). Modelling these two separately leads to a disentangled representation.
  In contrast, DDSP specifies the amplitude for each harmonic component directly resulting in entanglement and redundancy between these amplitudes. Furthermore, it remains uncertain whether the amplitudes ${a}_{k}^{t}$ could be effectively controlled and encoded by the brain. In our approach, we explicitly model the formant filters and fundamental frequency, which possess clear physical interpretations and are likely to be directly controlled by the brain. Our representation also enables a more robust and direct estimation of the pitch.

#### Speaker-specific synthesizer parameters

##### Prototype filters

Instead of using a predetermined prototype formant filter shape, for example, a standard Gaussian function, we learn a speaker-dependent prototype filter for each formant to allow more expressive and flexible formant filter shapes. We define the prototype filter *G*_*i*_(*f*) of the *i*th formant as a piecewise linear function, linearly interpolated from *g*_*i*_[*m*], *m* = 1, …, *M*, with the amplitudes of the filter at *M* being uniformly sampled frequencies in the range [0, *f*_max_]. We constrain *g*_*i*_[*m*] to increase and then decrease monotonically so that *G*_*i*_(*f*) is unimodal and has a single peak value of 1. Given *g*_*i*_[*m*], *m* = 1, …, *M*, we can determine the peak frequency ${f}_{i}^{\;{{{\rm{proto}}}}}$ and the half-power bandwidth ${b}_{i}^{{{{\rm{proto}}}}}$ of *G*_*i*_(*f*).

The prototype parameters *g*_*i*_[*m*], *m* = 1, …, *M* of each formant filter are time-invariant and are determined during the auto-encoder training. Compared with ref. ^29^, we increase *M* from 20 to 80 to enable more expressive formant filters, essential for synthesizing male speakers’ voices.

We similarly learn a prototype filter for the broadband filter *G*_*û*_(*f*) for the unvoiced component, which is specified by *M* parameters *g*_*û*_(*m*).

##### Background noise

The recorded sound typically contains background noise. We assume that the background noise is stationary and has a specific frequency distribution, depending on the speech recording environment. This frequency distribution *B*(*f*) is described by *K* parameters, where *K* is the number of frequency bins (*K* = 256 for females and 512 for males). The *K* parameters are also learned during auto-encoder training. The background noise is added to the mixed speech components to generate the final speech spectrogram.

To summarize, our speech synthesizer has the following learnable parameters: the *M* = 80 prototype filter parameters for each of the *N* = 6 formant filters and the broadband filters (totalling *M*(*N* + 1) = 560), the three parameters *f*_*θ*_, *a* and *b*_0_ relating the centre frequency and bandwidth for the formant filters (totalling 18), and *K* parameters for the background noise (256 for female and 512 for male). The total number of parameters for female speakers is 834, and that for male speakers is 1,090. Note that these parameters are speaker-dependent but time-independent, and they can be learned together with the speech encoder during the training of the speech-to-speech auto-encoder, using the speaker’s speech only.

### Speech encoder

The speech encoder extracts a set of (18) speech parameters at each time point from a given spectrogram, which are then fed to the speech synthesizer to reproduce the spectrogram.

We use a simple network architecture for the speech encoder, with temporal convolutional layers and multilayer perceptron (MLP) across channels at the same time point, as shown in Fig. 6a. We encode pitch ${f}_{0}^{\;t}$ by combining features generated from linear and Mel-scale spectrograms. The other 17 speech parameters are derived by applying temporal convolutional layers and channel MLP to the linear-scale spectrogram. To generate formant filter centre frequencies ${f}_{i = 1\,{{{\rm{to}}}}\,6}^{\;t}$, broadband unvoice filter frequency ${f}_{\hat{u}}^{\;t}$ and pitch ${f}_{0}^{\;t}$, we use sigmoid activation at the end of the corresponding channel MLP to map the output to [0, 1], and then de-normalize it to real values by scaling [0, 1] to predefined [*f*_min_, *f*_max_]. The [*f*_min_, *f*_max_] values for each frequency parameter are chosen based on previous studies^54–57^. Our compact speech parameter space facilitates stable and easy training of our speech encoder. Models were coded using PyTorch version 1.21.1 in Python.

### ECoG decoder

In this section we present the design details of three ECoG decoders: the 3D ResNet ECoG decoder, the 3D Swin transformer ECoG decoder and the LSTM ECoG decoder. The models were coded using PyTorch version 1.21.1 in Python.

#### 3D ResNet ECoG decoder

This decoder adopts the ResNet architecture^23^ for the feature extraction backbone of the decoder. Figure 6c illustrates the feature extraction part. The model views the ECoG input as 3D tensors with spatiotemporal dimensions. In the first layer, we apply only temporal convolution to the signal from each electrode, because the ECoG signal exhibits more temporal than spatial correlations. In the subsequent parts of the decoder, we have four residual blocks that extract spatiotemporal features using 3D convolution. After downsampling the electrode dimension to 1 × 1 and the temporal dimension to *T*/16, we use several transposed Conv layers to upsample the features to the original temporal size *T*. Figure 6b shows how to generate the different speech parameters from the resulting features using different temporal convolution and channel MLP layers. The temporal convolution operation can be causal (that is, using only past and current samples as input) or non-causal (that is, using past, current and future samples), leading to causal and non-causal models.

#### 3D Swin Transformer ECoG decoder

Swin Transformer^24^ employs the window and shift window methods to enable self-attention of small patches within each window. This reduces the computational complexity and introduces the inductive bias of locality. Because our ECoG input data have three dimensions, we extend Swin Transformer to three dimensions to enable local self-attention in both temporal and spatial dimensions among 3D patches. The local attention within each window gradually becomes global attention as the model merges neighbouring patches in deeper transformer stages.

Figure 6d illustrates the overall architecture of the proposed 3D Swin Transformer. The input ECoG signal has a size of *T* × *H* × *W*, where *T* is the number of frames and *H* × *W* is the number of electrodes at each frame. We treat each 3D patch of size 2 × 2 × 2 as a token in the 3D Swin Transformer. The 3D patch partitioning layer produces ${\frac{T}{2}\times \frac{H}{2}\times \frac{W}{2}}$ 3D tokens, each with a 48-dimensional feature. A linear embedding layer then projects the features of each token to a higher dimension *C* (=128).

The 3D Swin Transformer comprises three stages with two, two and six layers, respectively, for LD participants and four stages with two, two, six and two layers for HB participants. It performs 2 × 2 × 2 spatial and temporal downsampling in the patch-merging layer of each stage. The patch-merging layer concatenates the features of each group of 2 × 2 × 2 temporally and spatially adjacent tokens. It applies a linear layer to project the concatenated features to one-quarter of their original dimension after merging. In the 3D Swin Transformer block, we replace the multi-head self-attention (MSA) module in the original Swin Transformer with the 3D shifted window multi-head self-attention module. It adapts the other components to 3D operations as well. A Swin Transformer block consists of a 3D shifted window-based MSA module followed by a feedforward network (FFN), a two-layer MLP. Layer normalization is applied before each MSA module and FFN, and a residual connection is applied after each module.

Consider a stage with *T* × *H* × *W* input tokens. If the 3D window size is *P* × *M* × *M*, we partition the input into ${\lceil \frac{T}{P}\rceil \times \lceil \frac{H}{M}\rceil \times \lceil \frac{W}{M}\rceil}$ non-overlapping 3D windows evenly. We choose *P* = 16, *M* = 2. We perform the multi-head self-attention within each 3D window. However, this design lacks connection across adjacent windows, which may limit the representation power of the architecture. Therefore, we extend the shifted 2D window mechanism of the Swin Transformer to shifted 3D windows. In the second layer of the stage, we shift the window by $\left({\frac{P}{2},\,\frac{M}{2},\,\frac{M}{2}}\right)$ tokens along the temporal, height and width axes from the previous layer. This creates cross-window connections for the self-attention module. This shifted 3D window design enables the interaction of electrodes with longer spatial and temporal distances by connecting neighbouring tokens in non-overlapping 3D windows in the previous layer.

The temporal attention in the self-attention operation can be constrained to be causal (that is, each token only attends to tokens temporally before it) or non-causal (that is, each token can attend to tokens temporally before or after it), leading to the causal and non-causal models, respectively.

#### LSTM decoder

The decoder uses the LSTM architecture^25^ for the feature extraction in Fig. 6e. Each LSTM cell is composed of a set of gates that control the flow of information: the input gate, the forget gate and the output gate. The input gate regulates the entry of new data into the cell state, the forget gate decides what information is discarded from the cell state, and the output gate determines what information is transferred to the next hidden state and can be output from the cell.

In the LSTM architecture, the ECoG input would be processed through these cells sequentially. For each time step *T*, the LSTM would take the current input *x*_*t*_ and the previous hidden state *h*_*t* − 1_ and would produce a new hidden state *h*_*t*_ and output *y*_*t*_. This process allows the LSTM to maintain information over time and is particularly useful for tasks such as speech and neural signal processing, where temporal dependencies are critical. Here we use three layers of LSTM and one linear layer to generate features to map to speech parameters. Unlike 3D ResNet and 3D Swin, we keep the temporal dimension unchanged across all layers.

### Model training

#### Training of the speech encoder and speech synthesizer

As described earlier, we pre-train the speech encoder and the learnable parameters in the speech synthesizer to perform a speech-to-speech auto-encoding task. We use multiple loss terms for the training. The modified multi-scale spectral (MSS) loss is inspired by ref. ^53^ and is defined as

$${L}_{\rm{MSS}}({\widehat{S}}^{t}{(\;f\;)},\,{S}^{t}{(\;f\;)})={L}({\widehat{S}}^{t}{(\;f\;)},\,{S}^{t}{(\;f\;)})+{L}({\widehat{S}}_{{{{\rm{mel}}}}}^{t}{(\;f\;)},\,{S}_{{{{\rm{mel}}}}}^{t}{(\;f\;)})$$

with

$${L(x,\,y)}={\left\Vert x-y\right\Vert }_{1}+{\left\Vert \log x-\log y\right\Vert }_{1}$$

Here, *S*^*t*^(*f*) denotes the ground-truth spectrogram and ${\widehat{S}}^{t}{(\;f\;)}$ the reconstructed spectrogram in the linear scale, ${S}_{{{{\rm{mel}}}}}^{t}{(\;f\;)}$ and ${\widehat{S}}_{{{{\rm{mel}}}}}^{t}{(\;f\;)}$ are the corresponding spectrograms in the Mel-frequency scale. We sample the frequency range [0, 8,000 Hz] with *K* = 256 bins for female participants. For male participants, we set *K* = 512 because they have lower *f*_0_, and it is better to have a higher resolution in frequency.

To improve the intelligibility of the reconstructed speech, we also introduce the STOI loss by implementing the STOI+ metric^26^, which is a variation of the original STOI metric^8,22^. STOI+^26^ discards the normalization and clipping step in STOI and has been shown to perform best among intelligibility evaluation metrics. First, a one-third octave band analysis^22^ is performed by grouping Discrete Fourier transform (DFT) bins into 15 one-third octave bands with the lowest centre frequency set equal to 150 Hz and the highest centre frequency equal to ~4.3 kHz. Let ${\hat{x}(k,\,m)}$ denote the *k*th DFT bin of the *m*th frame of the ground-truth speech. The norm of the *j*th one-third octave band, referred to as a time-frequency (TF) unit, is then defined as

$${X}_{j}{(m)}={\sqrt{\mathop{\sum }\limits_{k={k}_{1}(\;j)}^{{k}_{2}(\;j)-1}| \hat{x}(k,\,m){| }^{2}}}$$

where *k*_1_(*j*) and *k*_2_(*j*) denote the one-third octave band edges rounded to the nearest DFT bin. The TF representation of the processed speech ${\hat{y}}$ is obtained similarly and denoted by *Y*_*j*_(*m*). We then extract the short-time temporal envelopes in each band and frame, denoted *X*_*j*, *m*_ and *Y*_*j*, *m*_, where ${X}_{j,\,m}={\left[{X}_{j}{(m-N+1)},\,{X}_{j}{(m-N+2)},\,\ldots ,\,{X}_{j}{(m)}\right]}^{\rm{T}}$, with *N* = 30. The STOI+ metric is the average of the PCC *d*_*j*, *m*_ between *X*_*j*, *m*_ and *Y*_*j*, *m*_, overall *j* and *m* (ref. ^26^):

$${\rm{STOI}}_{\rm{plus}}={\frac{1}{JM}\mathop{\sum}\limits_{j,\,m}{d}_{j,\,m}}$$

We use the negative of the STOI+ metric as the STOI loss:

$${L}_{\rm{STOI}}=-{\rm{STOI}}_{\rm{plus}}$$

where *J* and *M* are the total numbers of frequency bins (*J* = 15) and frames, respectively. Note that *L*_STOI_ is differentiable with respect to ${\widehat{S}}^{t}{(\;f\;)}$, and thus can be used to update the model parameters generating the predicted spectrogram ${\widehat{S}}^{t}{(\;f\;)}$.

To further improve the accuracy for estimating the pitch ${\widetilde{f}}_{0}^{\;t}$ and formant frequencies ${\widetilde{f}}_{{{{\rm{i}}}} = {1}\,{{{\rm{to}}}}\,4}^{\;t}$, we add supervisions to them using the formant frequencies extracted by the Praat method^50^. The supervision loss is defined as

$${L}_{{{{\rm{supervision}}}}}={\left\Vert\; {\widetilde{f}}_{0}^{\;t}-{f}_{0}^{\;t}\right\Vert }_{2}^{2}+\mathop{\sum }\limits_{i=1}^{4}{\beta }_{i}{\left\Vert\; {\widetilde{f}}_{i}^{\;t}-{f}_{i}^{\;t}\right\Vert }_{2}^{2}$$

where the weights *β*_*i*_ are chosen to be *β*_1_ = 0.1, *β*_2_ = 0.06, *β*_3_ = 0.03 and *β*_4_ = 0.02, based on empirical trials. The overall training loss is defined as

$${L}={L}_{{{{\rm{MSS}}}}}+{\lambda }_{1}{L}_{{{{\rm{STOI}}}}}+{\lambda }_{2}{L}_{{{{\rm{supervision}}}}}$$

where the weighting parameters *λ*_*i*_ are empirically optimized to be *λ*_1_ = 1.2 and *λ*_2_ = 0.1 through testing the performances on three hybrid-density participants with different parameter choices.

#### Training of the ECoG decoder

With the reference speech parameters generated by the speech encoder and the target speech spectrograms as ground truth, the ECoG decoder is trained to match these targets. Let us denote the decoded speech parameters as ${\widetilde{C}}_{j}^{\;t}$, and their references as ${C}_{j}^{\;t}$, where *j* enumerates all speech parameters fed to the speech synthesizer. We define the reference loss as

$${L}_{{{{\rm{reference}}}}}={\mathop{\sum}\limits_{j}{\lambda }_{j}{\left\Vert {\widetilde{C}}_{j}^{t}-{C}_{j}^{t}\right\Vert }_{2}^{2}}$$

where weighting parameters *λ*_*j*_ are chosen as follows: voice weight *λ*_*α*_ = 1.8, loudness *λ*_*L*_ = 1.5, pitch ${\lambda }_{{f}_{0}}={0.4}$, formant frequencies ${\lambda }_{{f}_{1}}={3},\,{\lambda }_{{f}_{2}}={1.8},\,{\lambda }_{{f}_{3}}={1.2},\,{\lambda }_{{f}_{4}}={0.9},\,{\lambda }_{{f}_{5}}={0.6},\,{\lambda }_{{f}_{6}}={0.3}$, formant amplitudes ${\lambda }_{{a}_{1}}={4},\,{\lambda }_{{a}_{2}}={2.4},\,{\lambda }_{{a}_{3}}={1.2},\,{\lambda }_{{a}_{4}}={0.9},\,{\lambda }_{{a}_{5}}={0.6},\,{\lambda }_{{a}_{6}}={0.3}$, broadband filter frequency ${\lambda }_{{f}_{\hat{u}}}={10}$, amplitude ${\lambda }_{{a}_{\hat{u}}}={4}$, bandwidth ${\lambda }_{{b}_{\hat{u}}}={4}$. Similar to speech-to-speech auto-encoding, we add supervision loss for pitch and formant frequencies derived by the Praat method and use the MSS and STOI loss to measure the difference between the reconstructed spectrograms and the ground-truth spectrogram. The overall training loss for the ECoG decoder is

$${L}={L}_{{{{\rm{MSS}}}}}+{\lambda }_{1}{L}_{{{{\rm{STOI}}}}}+{\lambda }_{2}{L}_{{{{\rm{supervision}}}}}+{\lambda }_{3}{L}_{{{{\rm{reference}}}}}$$

where weighting parameters *λ*_*i*_ are empirically optimized to be *λ*_1_ = 1.2, *λ*_2_ = 0.1 and *λ*_3_ = 1, through the same parameter search process as described for training the speech encoder.

We use the Adam optimizer^58^ with hyper-parameters *lr* = 10^−3^, *β*_1_ = 0.9 and *β*_2_ = 0.999 to train both the auto-encoder (including the speech encoder and speech synthesizer) and the ECoG decoder. We train a separate set of models for each participant. As mentioned earlier, we randomly selected 50 out of 400 trials per participant as the test data and used the rest for training.

### Evaluation metrics

In this Article, we use the PCC between the decoded spectrogram and the actual speech spectrogram to evaluate the objective quality of the decoded speech, similar to refs. ^8,18,59^.

We also use STOI+^26^, as described in Methods section Training of the ECoG decoder to measure the intelligibility of the decoded speech. The STOI+ value ranges from −1 to 1 and has been reported to have a monotonic relationship with speech intelligibility.

### Contribution analysis with the occlusion method

To measure the contribution of the cortex region under each electrode to the decoding performance, we adopted an occlusion-based method that calculates the change in the PCC between the decoded and the ground-truth spectrograms when an electrode signal is occluded (that is, set to zeros), as in ref. ^29^. This method enables us to reveal the critical brain regions for speech production. We used the following notations: *S*^*t*^(*f*), the ground-truth spectrogram; ${\hat{{{{{S}}}}}}^{t}{(\;f\;)}$, the decoded spectrogram with ‘intact’ input (that is, all ECoG signals are used); ${\hat{{{{{S}}}}}}_{i}^{t}{(\;f\;)}$, the decoded spectrogram with the *i*th ECoG electrode signal occluded; *r*(⋅, ⋅), correlation coefficient between two signals. The contribution of *i*th electrode for a particular participant is defined as

$${{{{\rm{C}}}}}^{i}={{{{\rm{Mean}}}}\left\{{r}\left({{{{\rm{S}}}}}^{t}{(\;f\;)},\,{\hat{{{{\rm{S}}}}}}^{t}{(\;f\;)}\right)-{r}\left({{{{\rm{S}}}}}^{t}{(\;f\;)},\,{\hat{{{{\rm{S}}}}}}_{i}^{t}{(\;f\;)}\right)\right\}}$$

where Mean{⋅} denotes averaging across all testing trials of the participant.

We generate the contribution map on the standardized Montreal Neurological Institute (MNI) brain anatomical map by diffusing the contribution of each electrode of each participant (with a corresponding location in the MNI coordinate) into the adjacent area within the same anatomical region using a Gaussian kernel and then averaging the resulting map from all participants. To account for the non-uniform density of the electrodes in different regions and across the participants, we normalize the sum of the diffused contribution from all the electrodes at each brain location by the total number of electrodes in the region across all participants.

We estimate the noise level for the contribution map to assess the significance of our contribution analysis. To derive the noise level, we train a shuffled model for each participant by randomly pairing the mismatched speech segment and ECoG segment in the training set. We derive the average contribution map from the shuffled models for all participants using the same occlusion analysis as described earlier. The resulting contribution map is used as the noise level. Contribution levels below the noise levels at corresponding cortex locations are assigned a value of 0 (white) in Fig. 4.

### Reporting summary

Further information on research design is available in the Nature Portfolio Reporting Summary linked to this Article.

## Supplementary information

Supplementary Information Supplementary Figs. 1–10, Table 1 and audio files list.

Reporting Summary

Supplementary Audio 1 Example original and decoded audios for eight words.

Supplementary Audio 2 Example original and decoded words from low density participants.

Supplementary Audio 3 Example original and decoded words from hybrid density participants.

Supplementary Audio 4 Example original and decoded words from left hemisphere low density participants.

Supplementary Audio 5 Example original and decoded words from right hemisphere low density participants.

## Source data

Source Data Fig. 2 Data for Fig, 2a,b,d,e,f.

Source Data Fig. 3 Data for Fig, 3a,c,d.

## Data Availability

The data of one participant who consented to the release of the neural and audio data are publicly available through Mendeley Data at https://data.mendeley.com/datasets/fp4bv9gtwk/2 (ref. ^60^). Although all participants consented to share their data for research purposes, not all participants agreed to share their audio publicly. Given the sensitive nature of audio speech data we will share data with researchers that directly contact the corresponding author and provide documentation that the data will be strictly used for research purposes and will comply with the terms of our study IRB. Source data are provided with this paper.

## Code availability

The code is available at https://github.com/flinkerlab/neural_speech_decoding (10.5281/zenodo.10719428)^61^.
