# Supplementary material for: A neural speech decoding framework leveraging deep learning and speech synthesis
Source: Nat Mach Intell. 2024 Apr 8;6(4):467–80. doi: 10.1038/s42256-024-00824-8 (PMC12965882; doi:10.1038/s42256-024-00824-8)
Supplement: Supplementary file 1 — Supplementary Figs. 1–10, Table 1 and audio files list. [file 42256_2024_824_MOESM1_ESM.pdf]

nature machine intelligence

Article

https://doi.org/10.1038/s42256-024-00824-8

A neural speech decoding framework leveraging deep learning and speech synthesis

In the format provided by the authors and unedited

CONTENTS

1 STOI+ as evaluation metrics 2

2 Non-causal model evaluation and visualization 3

3 Comparison of ResNet and 3D SWIN across patients with LD, HB, and LD-in-HB 4

4 Comparison of ResNet and 3D SWIN across patients with left and right hemisphere 5

5 Acoustic contamination analysis 6

6 Model inference time evaluation 7

7 Electrodes placement visualization 8

8 Contribution analysis supplementary figures 10

9 Comparison of proposed model and GAN approach in the previous study 11

10 Supplementary Audio Files 12

References 13

1. STOI+ AS EVALUATION METRICS

The main manuscript presented the decoding performance in terms of the PCC. Here we present additional evaluations using the STOI+ metric, which has a better correlation with the intelligibility of decoded speech. We also present additional results obtained with the non-causal model.

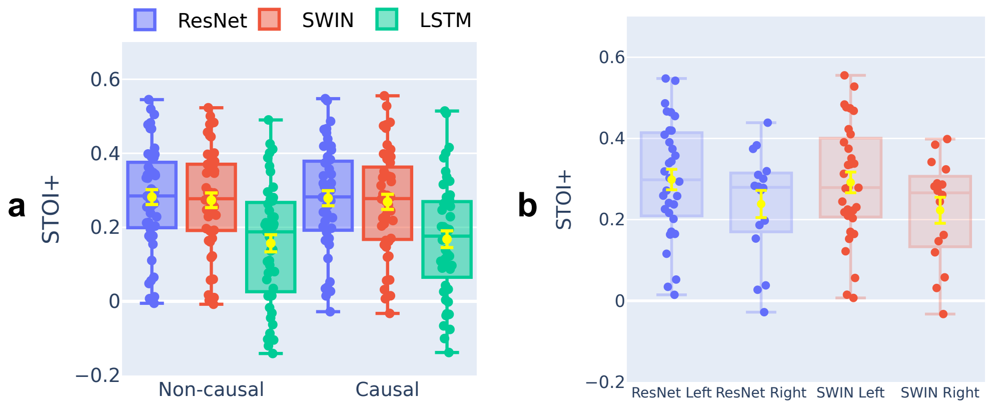

Fig. S1. | **Comparison of decoding STOI+ under different settings of the 3D ResNet, 3D SWIN, and LSTM models.** **a,** Performance of ResNet, SWIN, and LSTM models with non-causal and causal operations across all participants (N=48; 43 low-density ECoG grids and 5 hybrid density grids). The STOI+ between the original and decoded spectrogram is evaluated on the held-out testing set and shown for each participant. Each data point corresponds to a participant's average PCC across all testing trials. The boxplot represents the median, 25th and 75th quantiles across participants, and the yellow error bar denotes the mean and standard error of the mean. As with PCC (Fig. 2a in the manuscript), ResNet and SWIN models perform similarly, but the LSTM model is significantly worse. **b,** STOI+ comparison between left and right hemisphere participants, using causal ResNet and SWIN models. No statistically significant decoding performance differences exist between left (N=32) and right (N=16) hemisphere participants (ResNet independent t-test, p=0.166; SWIN independent t-test, p=0.114), although the left hemisphere participants have slightly greater mean STOI+. All box plots depict the median (horizontal line inside box), 25th and 75th percentiles (box), 25th or 75th percentiles $\pm 1.5\times$ interquartile range (whiskers). The yellow error bars denote the mean $\pm$ SEM.

2. NON-CAUSAL MODEL EVALUATION AND VISUALIZATION

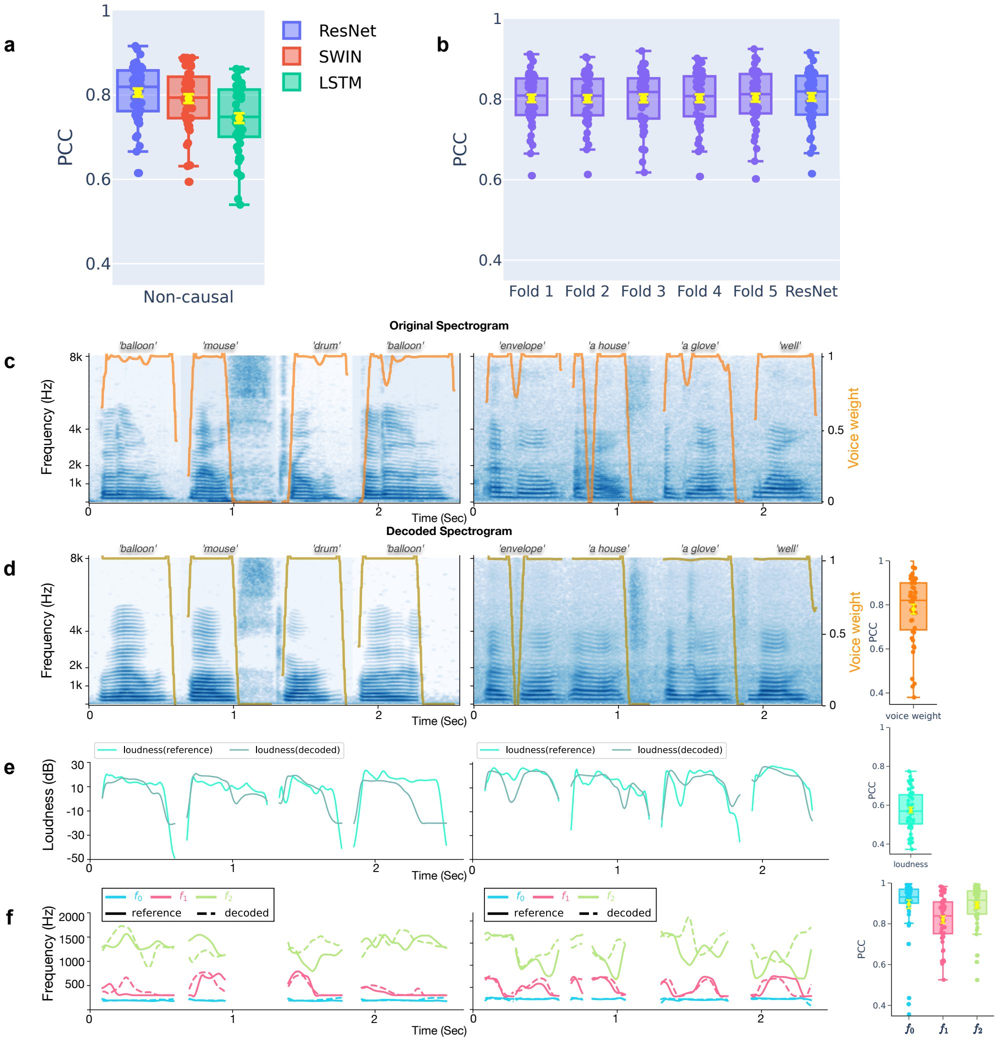

Fig. S2. | **Decoding performance comparing the original and decoded spectrograms across non-causal models.** **a,** PCC between the original and decoded spectrograms by non-causal ResNet, 3D SWIN, and LSTM models for all participants (N=48), including 43 participants with LD ECoG grids and 5 participants with HB grids, evaluated on the held-out testing set, which contains different speech trials from the training set but include overlapping words. **b,** ResNet model PCC on unseen words during training from a 5-fold cross-validation study where the training and validation sets in each fold have non-overlapping words. The performance across five folds is similar to randomly selected test trials. **c-f,** Example decoded spectrograms and speech parameters by the non-causal ResNet model for four words each from two participants and PCC between the decoded and reference speech parameters across all participant trials. **c,d,** Comparison of original (**c**) and decoded (**d**) spectrograms. The orange curves overlaid on the spectrograms in **c** and **d** show the reference voice weight generated by the speech encoder and the decoded voice weight by the ECoG decoder, respectively. The box plot in **d** shows the PCC between the decoded and reference voice weight for all participants (N=48). **e,** Decoded loudness parameter compared to reference loudness parameter. The box plot shows the PCC of the decoded and reference loudness parameters (N=48). **f,** Comparison of the decoded and reference f_0 and f_1, f_2 (N=48). The results achieved with non-causal models follow a very similar trend as those obtained with causal models, shown in Fig. 2f. All box plots depict the median (horizontal line inside box), 25th and 75th percentiles (box), 25th or 75th percentiles $\pm 1.5\times$ interquartile range (whiskers). The yellow error bars denote the mean $\pm$ SEM.

3. COMPARISON OF RESNET AND 3D SWIN ACROSS PATIENTS WITH LD, HB, AND LD-IN-HB

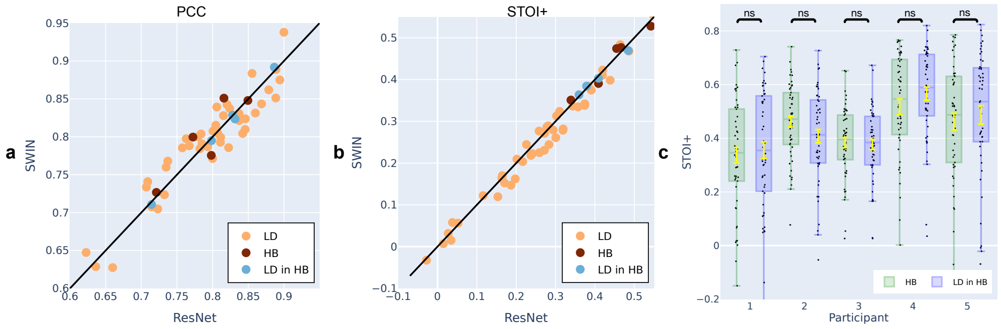

Fig. S3. | **Comparison of decoding PCC and STOI+ of the causal 3D ResNet and 3D SWIN models for the same participant across participants with hybrid-density (HB, N=5), low-density (LD, N=43), and only LD-in-HB ECoG grids.** In terms of PCC (**a**), both models have similar decoding performances on both HB, LD, and LD-in-HB participants. In terms of STOI+ (**b**), both models have slightly better performance on the HB participants, and the ResNet has similar performance as the SWIN model for both HB and LD participants. The LD-in-HB (N=5) and HB participants (N=5) have very similar performances in both PCC and STOI+ (**c**). The decoding STOI+ by the ResNet model for HB participants (N=5) when all electrodes are used vs when only LD-in-HB electrodes (N=5) are considered. There are no statistically significant differences for all participants (Wilcoxon two-sided signed-rank test, p-value = 0.626, 0.146, 0.881, 0.058, 0.414). In 3 out of 5 participants, using LD-in-HB electrodes even gives us higher STOI+ compared with using HB only. Box plot depicts the median (horizontal line inside box), 25th and 75th percentiles (box), 25th or 75th percentiles $\pm 1.5\times$ interquartile range (whiskers). The yellow error bars denote the mean $\pm$ SEM. Distributions were compared with each other as indicated using the Wilcoxon two-sided signed-rank test. $ns > 0.05$.

4. COMPARISON OF RESNET AND 3D SWIN ACROSS PATIENTS WITH LEFT AND RIGHT HEMISPHERE

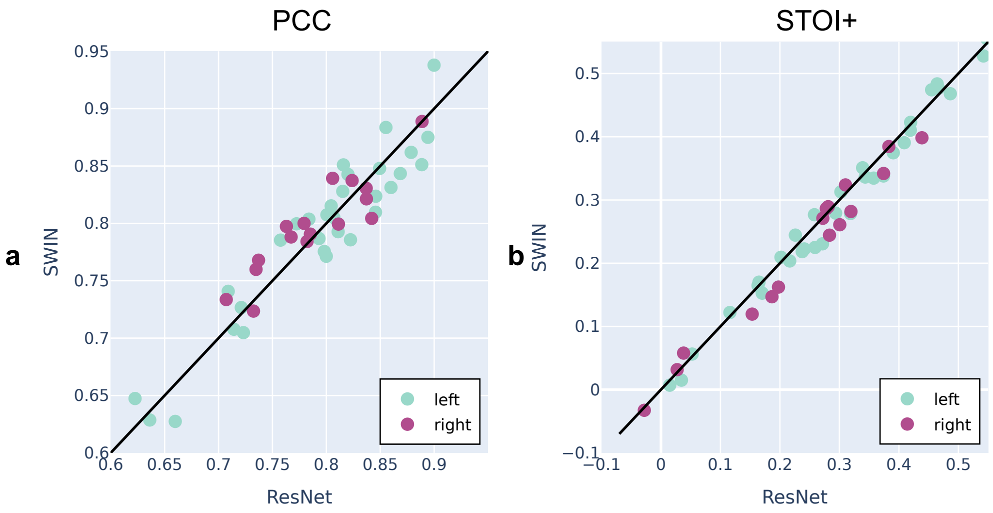

Fig. S4. | **Comparison of decoding PCC and STOI+ of the causal 3D ResNet and 3D SWIN models for the same participant across participants with right hemisphere (N=16) or left hemisphere (N=32) grids** (**a**) In terms of PCC, both models have similar decoding performances on the left and right hemispheres (ResNet independent t-test, p=0.623; SWIN independent t-test, p=0.968). (**b**) In terms of STOI+, both models have slightly better performance for the left hemisphere decoding, and the ResNet model has better performance for both left and right hemisphere decoding. Still, the difference is not statistically significant. (ResNet independent t-test, p=0.166; independent t-test, p=0.114)

5. ACOUSTIC CONTAMINATION ANALYSIS

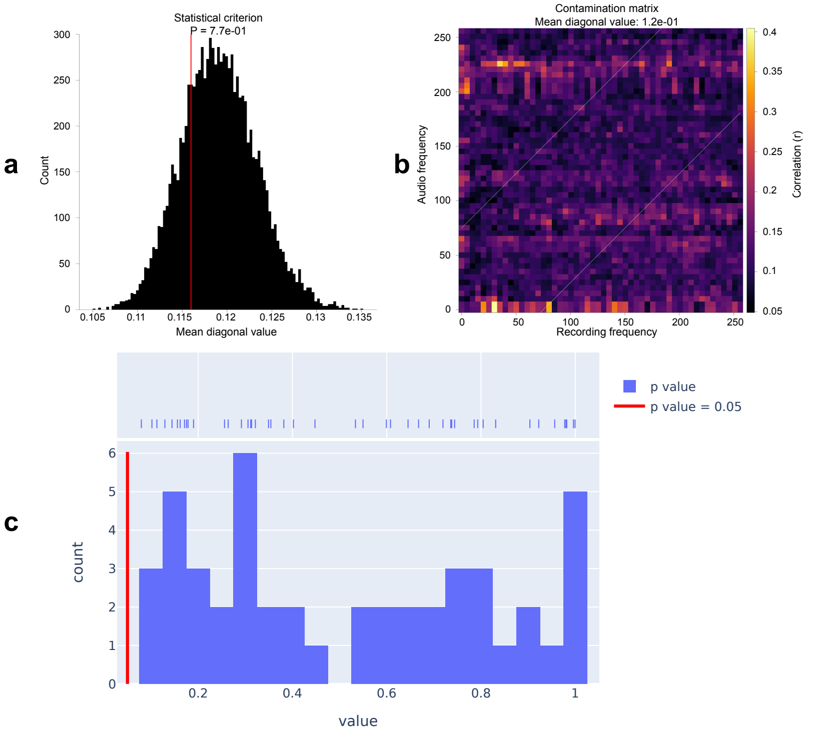

Fig. S5. Acoustic contamination evaluation. We calculate the correlation matrix between frequency components in the audio signal and the neural ECoG recordings and obtain the statistical assessment of contamination. The analysis follows the protocol reported in([1]). **(a),** Statistical contamination assessment in one participant (LD43). The mean of the diagonal of the contamination matrix (the vertical rad bar) and histogram of such value in 10000 shuffled contamination matrices. ECoG recordings' criterion (P= 0.77) rejects the null hypothesis that neural recordings have acoustic contamination. For each value of correlations between the neural and the audio spectrograms r, a p-value was computed using Student's t-test to test the null hypothesis that r = 0. **(b),** The correlation matrix between frequency components in the audio signal and the neural ECoG recordings in one example participant. **(c),** We conduct contamination analysis across all participants (N=48). We plot the criterion (p-value) of each participant in the histogram. Every participant's analysis disproves the null hypothesis that neural recordings are contaminated acoustically.

6. MODEL INFERENCE TIME EVALUATION

				Time(ms)					
				ECoG-to-Spec		Spec-to-Wave		Total	
Device	Model	N_FFT	Causality	mean	std	mean	std	mean	std
GPU	SWIN 3D	256	anticausal	43.77	0.57	13.86	0.14	57.63	0.71
			**causal**	**45.22**	**0.62**	**13.91**	**0.12**	**59.13**	**0.74**
			noncausal	43.84	0.90	13.89	0.12	57.73	1.02
		512	anticausal	45.57	0.70	14.17	0.14	59.74	0.84
			**causal**	**46.78**	**0.62**	**14.09**	**0.13**	**60.87**	**0.75**
			noncausal	45.48	0.71	14.24	0.57	59.72	1.28
	LSTM	256	anticausal	28.29	0.87	15.36	4.80	43.65	5.67
			**causal**	**27.44**	**0.69**	**13.78**	**0.50**	**41.22**	**1.19**
			noncausal	27.91	0.37	13.66	0.12	41.57	0.49
		512	anticausal	28.91	0.57	13.88	0.20	42.79	0.77
			**causal**	**28.91**	**1.14**	**13.86**	**0.14**	**42.77**	**1.28**
			noncausal	29.10	0.62	13.88	0.35	42.98	0.97
	ResNet	256	anticausal	30.02	0.51	13.32	0.54	43.34	1.05
			**causal**	**30.70**	**0.53**	**13.29**	**0.56**	**43.99**	**1.09**
			noncausal	30.03	1.02	13.42	1.05	43.45	2.07
		512	anticausal	31.48	0.48	13.43	0.37	44.91	0.85
			**causal**	**32.45**	**0.47**	**13.52**	**0.33**	**45.97**	**0.80**
			noncausal	31.80	0.60	14.04	1.67	45.84	2.27

Table S1. Inference time of different model configurations on GPU device (RTX-3060). We did ECoG to Spectrogram and Spectrogram to Waveform separately.

7. ELECTRODES PLACEMENT VISUALIZATION

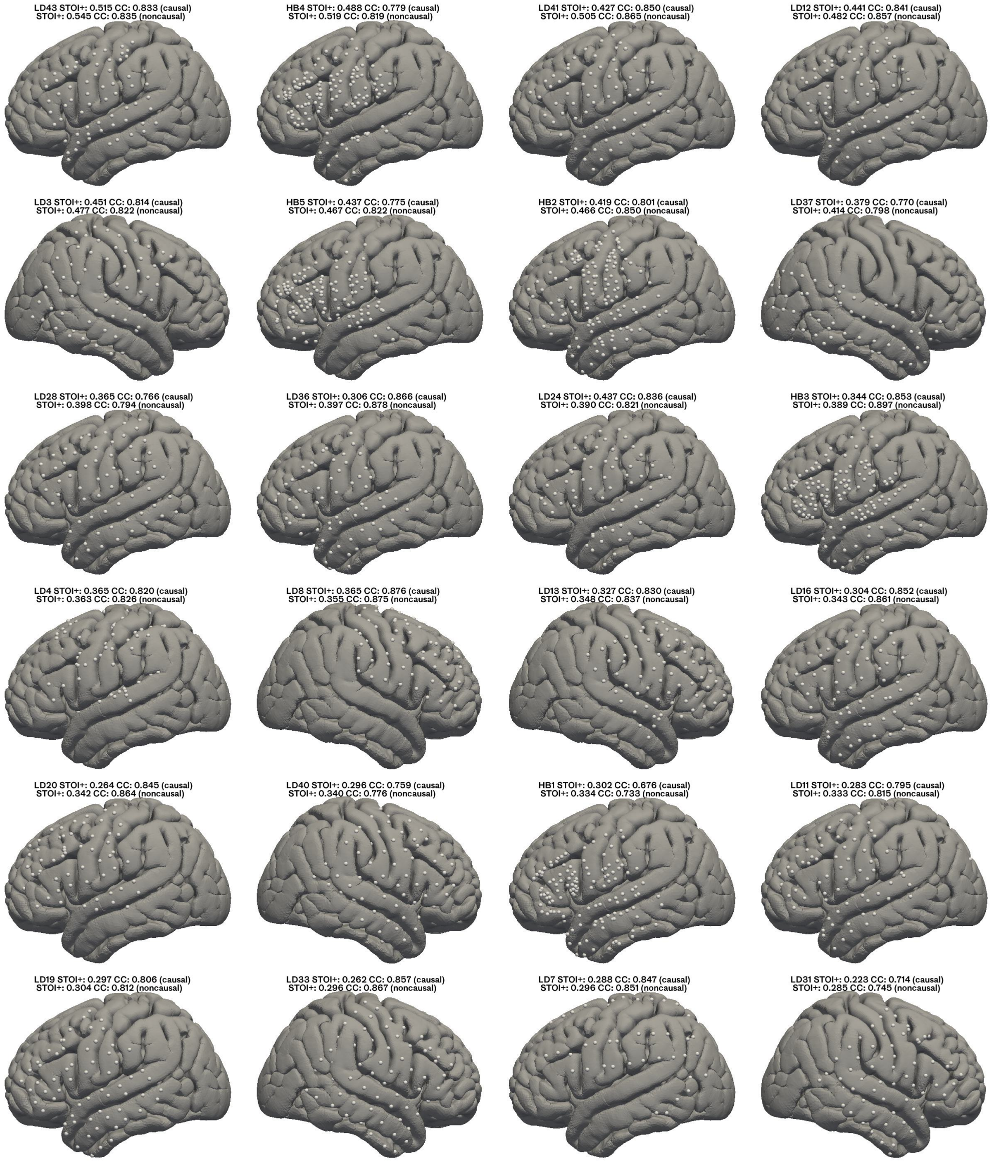

Fig. S6. | **Electrodes Placement of each Participant (first 24 participants)** We display each participant's electrode placement on MNI brain. The participants are ordered by the STOI+ value, and we report mean STOI+ and CC across test trials within each participant.

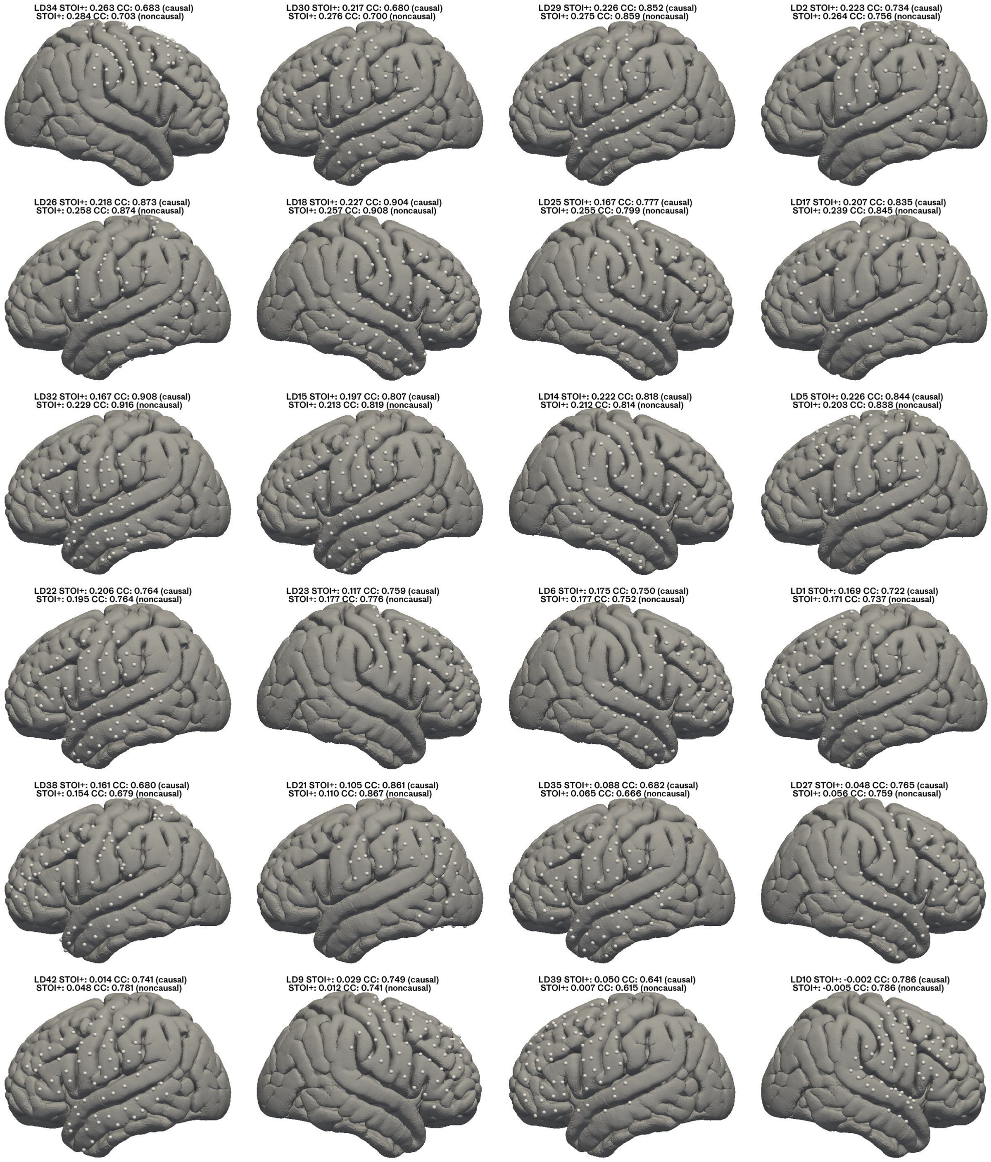

Fig. S7. | **Electrodes Placement of each Participant (second 24 participants)** We display each participant's electrode placement on MNI brain. The participants are ordered by the STOI+ value, and we report mean STOI+ and CC across test trials within each participant.

8. CONTRIBUTION ANALYSIS SUPPLEMENTARY FIGURES

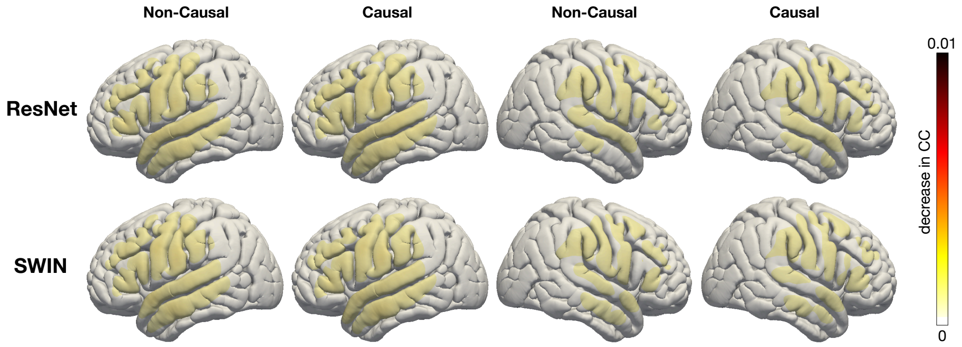

Fig. S8. | **Noise level of contribution analysis**. To derive the noise level, we trained a shuffled model for each participant by randomly pairing the mismatched speech segment and ECoG segment in the training set. We derive the average contribution map from the shuffled models for all participants (N=48) using the same occlusion analysis described in the Method section: Contribution Analysis Using the Occlusion Method. Contribution of each cortical location to the decoding result by both causal or non-causal random shuffled decoding models through an occlusion analysis. The contribution of each electrode region in each participant is projected onto the standardized Montreal Neurological Institute (MNI) brain anatomical map and then averaged over all participants. Each subplot shows the causal or non-causal contribution of different cortical locations (red indicates higher contribution while yellow indicates lower contribution). For visualization purposes, we normalize the contribution of each electrode location by the local grid density since we have multiple participants with non-uniform density. And we further normalize the value for visualization purposes.

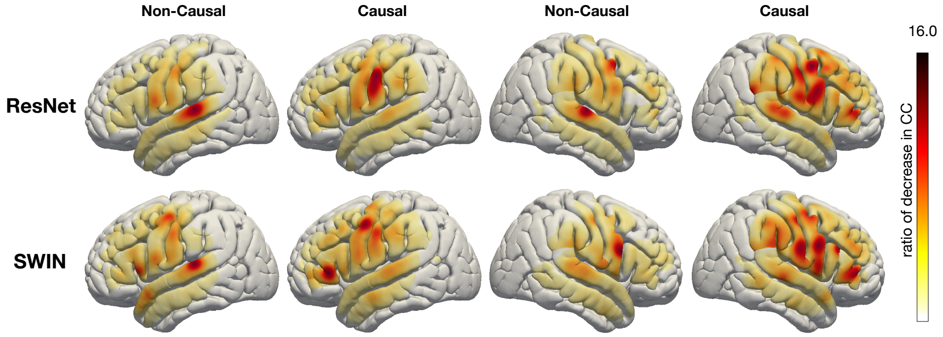

Fig. S9. | **Ratio of contribution analysis value vs the noise level of contribution analysis (N=48)**. Each subplot shows the causal or non-causal contribution of different cortical locations (red indicates higher contribution while yellow indicates lower contribution). For visualization purposes, we normalize the contribution of each electrode location by the local grid density since we have multiple participants with non-uniform density. And we further normalize the value for visualization purposes.

9. COMPARISON OF PROPOSED MODEL AND GAN APPROACH IN THE PREVIOUS STUDY

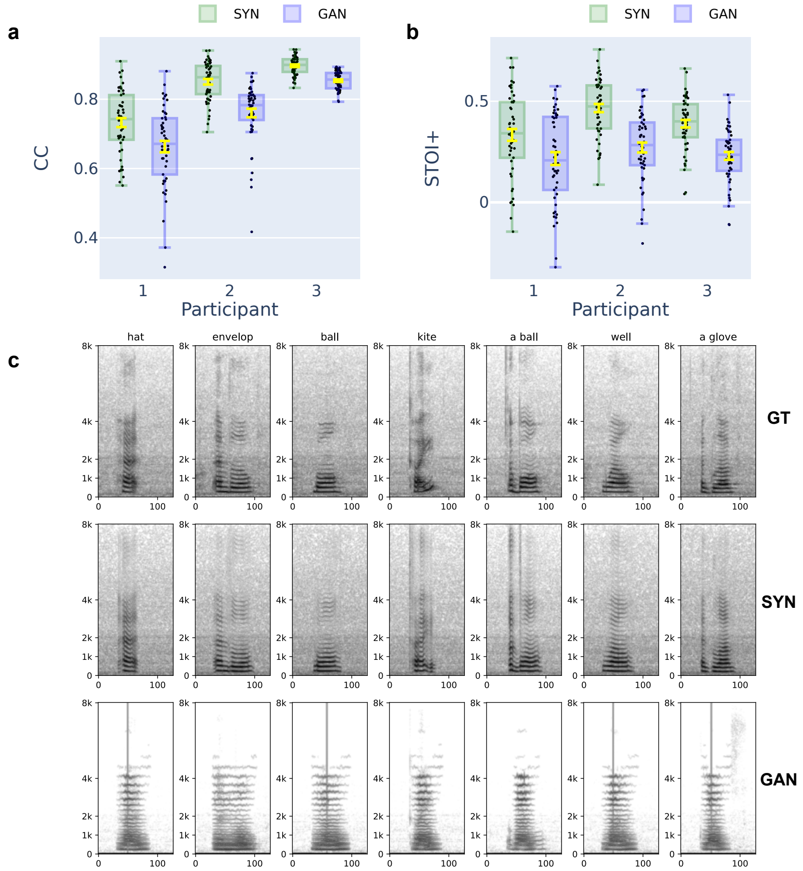

Fig. S10. | **Comparison of the current approach (SYN) using a ResNet for ECoG decoder and the proposed speech synthesizer with an approach (GAN) using a GAN-based speech synthesizer.** We applied a direct ECoG to spectrogram model (with WaveNet as a vocoder) developed and reported in [2] to speech production data in three of our HB participants. **a** and **b** shows the CC and STOI+ comparison of the two models. All box plots depict the median (horizontal line inside box), 25th and 75th percentiles (box), 25th or 75th percentiles $\pm 1.5\times$ interquartile range (whiskers). The yellow error bars denote the mean $\pm$ SEM. **c** Reconstructed spectrograms from decoded samples across the models. The upper row (GT) shows the ground truth spectrogram of the produced speech, the middle row (SYN) shows the decoded spectrogram using the ResNet ECoG decoder and our speech synthesizer, and the bottom row (GAN) shows the decoded spectrogram using the GAN approach. Our new approach (SYN) provides much closer (and well-formed) spectrograms, producing natural sound closer to the participant's voice in contrast to a robotic synthesis by the GAN approach.

10. SUPPLEMENTARY AUDIO FILES

The audio files are also stored in https://xc1490.github.io/nsd/ and in GitHub repo

- **GT**: ground truth audio files
  - HB_gt.wav: Hybrid-density participants ground truth audio
  - LD_gt.wav: Low-density participants ground truth audio
  - LEFT_gt.wav: Left hemisphere (LD) participants ground truth audio
  - RIGHT_gt.wav: Right hemisphere (LD) participants ground truth audio
- **PRED**: ResNet Decoder + Speech Synthesizer decoded speech from ECoG
  - HB_pred.wav: Hybrid-density participants ECoG decoded audio
  - LD_pred.wav: Low-density participants ECoG decoded audio
  - LEFT_pred.wav: Left hemisphere (LD) participants ECoG decoded audio
  - RIGHT_pred.wav: Right hemisphere (LD) participants ECoG decoded audio
- **Merge**: merge of ground truth and decoded audio
  - HB_merge.wav: Hybrid-density participants
  - LD_merge.wav: Low-density participants ECoG
  - LEFT_merge.wav: Left hemisphere (LD) participants
  - RIGHT_merge.wav: Right hemisphere (LD) participants

Audio files in ISBI_GAN

- wave_gt.wav: Hybrid-density participants ground truth audio
- wave_pred_gan.wav: Hybrid-density participants transfer-GAN decoded speech from ECoG
- wave_pred_syn.wav: Hybrid-density participants ResNet Decoder + Speech Synthesizer decoded speech from ECoG
- wave_merge.wav: merge of ground truth, ResNet decoded, and GAN decoded speech.

REFERENCES

1. P. Roussel, G. Le Godais, F. Bocquelet, M. Palma, J. Hongjie, S. Zhang, A.-L. Giraud, P. Mégevand, K. Miller, J. Gehrig *et al.*, "Observation and assessment of acoustic contamination of electrophysiological brain signals during speech production and sound perception," J. Neural Eng. **17**, 056028 (2020).
2. R. Wang, X. Chen, A. Khalilian-Gourtani, Z. Chen, L. Yu, A. Flinker, and Y. Wang, "Stimulus speech decoding from human cortex with generative adversarial network transfer learning," in *2020 IEEE 17th International Symposium on Biomedical Imaging (ISBI),* (IEEE, 2020), pp. 390–394.
