# Supplementary material for: A neural speech decoding framework leveraging deep learning and speech synthesis
Source: Nat Mach Intell. 2024 Apr 8;6(4):467–80. doi: 10.1038/s42256-024-00824-8 (PMC12965882; doi:10.1038/s42256-024-00824-8)
Supplement: Supplementary file 2 — Reporting Summary [file 42256_2024_824_MOESM2_ESM.pdf]

nature portfolio

Corresponding author(s): Adeen Flinker

Last updated by author(s): Dec 27, 2023

Reporting Summary

Nature Portfolio wishes to improve the reproducibility of the work that we publish. This form provides structure for consistency and transparency in reporting. For further information on Nature Portfolio policies, see our Editorial Policies and the Editorial Policy Checklist.

Statistics

For all statistical analyses, confirm that the following items are present in the figure legend, table legend, main text, or Methods section.

n/a	Confirmed	
☐	☒	The exact sample size (*n*) for each experimental group/condition, given as a discrete number and unit of measurement
☐	☒	A statement on whether measurements were taken from distinct samples or whether the same sample was measured repeatedly
☐	☒	The statistical test(s) used AND whether they are one- or two-sided<br>*Only common tests should be described solely by name; describe more complex techniques in the Methods section.*
☐	☒	A description of all covariates tested
☐	☒	A description of any assumptions or corrections, such as tests of normality and adjustment for multiple comparisons
☐	☒	A full description of the statistical parameters including central tendency (e.g. means) or other basic estimates (e.g. regression coefficient) AND variation (e.g. standard deviation) or associated estimates of uncertainty (e.g. confidence intervals)
☐	☒	For null hypothesis testing, the test statistic (e.g. *F*, *t*, *r*) with confidence intervals, effect sizes, degrees of freedom and *P* value noted<br>*Give P values as exact values whenever suitable.*
☒	☐	For Bayesian analysis, information on the choice of priors and Markov chain Monte Carlo settings
☒	☐	For hierarchical and complex designs, identification of the appropriate level for tests and full reporting of outcomes
☐	☒	Estimates of effect sizes (e.g. Cohen's *d*, Pearson's *r*), indicating how they were calculated

Our web collection on statistics for biologists contains articles on many of the points above.

Software and code

Policy information about availability of computer code

Data collection	MATLAB 2016a, Python 3.8
Data analysis	MATLAB 2016a, Python 3.8, Pytorch 1.21.1, Librosa 0.8.0, https://github.com/flinkerlab/neural_speech_decoding

For manuscripts utilizing custom algorithms or software that are central to the research but not yet described in published literature, software must be made available to editors and reviewers. We strongly encourage code deposition in a community repository (e.g. GitHub). See the Nature Portfolio guidelines for submitting code & software for further information.

Data

Policy information about availability of data

All manuscripts must include a data availability statement. This statement should provide the following information, where applicable:

- Accession codes, unique identifiers, or web links for publicly available datasets
- A description of any restrictions on data availability
- For clinical datasets or third party data, please ensure that the statement adheres to our policy

The data of one participant who consented to the release of the neural and audio data is publicly available through Mendeley Data https://data.mendeley.com/datasets/fp4bv9gtwk/. While all participants consented to share their data for research purposes, not all participants agreed to share their audio publicly. Given the sensitive nature of audio speech data we will share data with researchers that directly contact the corresponding author and provide documentation that the data will be strictly used for research purposes and will comply with the terms of our study IRB.

Human research participants

Policy information about studies involving human research participants and Sex and Gender in Research.

Reporting on sex and gender	Findings apply to two genders (Female and Male). Gender is considered in the study design and determined by self reporting. 26 Female and 22 Male participants' data are collected with their consent. The analysis for Female and Male participants are slightly different based on their acoustic characteristic differences.
Population characteristics	Participants were all diagnosed with epilepsy and required surgical treatment (a prerequisite for participation in human ECoG research). Due to this data collection setup, no covariates or participant properties could be controlled for.
Recruitment	Participants were recruited as part of their ongoing neurosurgical clinical care for Epilepsy. Once a patient was identified as undergoing surgical procedures they were first approached by their clinician to ask if they are interested and if a member of the research team could contact them. Prior to surgery, or post surgery at bedside, a member of the research team contacted the patient (after explicit consent they provided to the clinician) and explained the research. After research was explained, written and oral permission was obtained.<br>Patients were consented to participate in research only if they were clinical candidates for a two-staged neurosurgical procedure, had cognitive capacity to consent and provided approval to be approached for consent by their clinical provider. Since the participants' medical condition has had no impact on their ability to speak fluently, we do not expect there to be any selection bias and we believe them to be representative of general population.
Ethics oversight	The study was conducted under a New York University Grossman School of Medicine approved IRB.

Note that full information on the approval of the study protocol must also be provided in the manuscript.

Field-specific reporting

Please select the one below that is the best fit for your research. If you are not sure, read the appropriate sections before making your selection.

☒ Life sciences ☐ Behavioural & social sciences ☐ Ecological, evolutionary & environmental sciences

For a reference copy of the document with all sections, see nature.com/documents/nr-reporting-summary-flat.pdf

Life sciences study design

All studies must disclose on these points even when the disclosure is negative.

Sample size	The amount of speech data collected from each participant was dependent on their clinical treatment schedule and the experimental design. As explained in the manuscript each participant performed 5 auditory tasks and 400 trials are recorded. No sample size calculation was performed. A total of 48 neurosurgical patients that were implanted with electrocorticography electrodes were included in this study. Electrode implantation and location were guided solely by clinical requirements. Previous study used around 1-5 patients recordings. In this study we use a sample size a magnitude larger and the sample size should be sufficient.
Data exclusions	No data exclusions
Replication	Decoding performance was first done with one participant's data and then re-done with other 47 participants to confirm findings. The attempts to replicate were successful.
Randomization	The trials used to test decoding performances are randomly chosen. We also performed five fold cross validation to validate the decoding performance. There is no experimental group. We only have within subject decoding experiment.
Blinding	Blinding was not relevant for this study. The participants' speaking of words are not biased.

Reporting for specific materials, systems and methods

We require information from authors about some types of materials, experimental systems and methods used in many studies. Here, indicate whether each material, system or method listed is relevant to your study. If you are not sure if a list item applies to your research, read the appropriate section before selecting a response.

Materials & experimental systems

n/a	Involved in the study
☒	☐ Antibodies
☒	☐ Eukaryotic cell lines
☒	☐ Palaeontology and archaeology
☒	☐ Animals and other organisms
☒	☐ Clinical data
☒	☐ Dual use research of concern

Methods

n/a	Involved in the study
☒	☐ ChIP-seq
☒	☐ Flow cytometry
☒	☐ MRI-based neuroimaging
